# Insights into trait-association of selection signatures and adaptive eQTL in indigenous African cattle

**DOI:** 10.1186/s12864-024-10852-8

**Published:** 2024-10-19

**Authors:** Juliane Friedrich, Shuli Liu, Lingzhao Fang, James Prendergast, Pamela Wiener

**Affiliations:** 1 Division of Genetics and Genomics, The Roslin Institute and Royal (Dick) School of Veterinary Studies, University of Edinburgh, Midlothian, UK; 2 Westlake Laboratory of Life Sciences and Biomedicine, Hangzhou, Zhejiang China; 3 School of Life Sciences, Westlake University, Hangzhou, Zhejiang China; 4 Center for Quantitative Genetics and Genomics (QGG), Aarhus University, Aarhus, Denmark

**Keywords:** iHS, QTL, Gene expression, Environmental adaptation, Cattle GTEx

## Abstract

**Background:**

African cattle represent a unique resource of genetic diversity in response to adaptation to numerous environmental challenges. Characterising the genetic landscape of indigenous African cattle and identifying genomic regions and genes of functional importance can contribute to targeted breeding and tackle the loss of genetic diversity. However, pinpointing the adaptive variant and determining underlying functional mechanisms of adaptation remains challenging.

**Results:**

In this study, we use selection signatures from whole-genome sequence data of eight indigenous African cattle breeds in combination with gene expression and quantitative trait loci (QTL) databases to characterise genomic targets of artificial selection and environmental adaptation and to identify the underlying functional candidate genes. In general, the trait-association analyses of selection signatures suggest the innate and adaptive immune system and production traits as important selection targets. For example, a large genomic region, with selection signatures identified for all breeds except N’Dama, was located on BTA27, including multiple defensin *DEFB* coding-genes. Out of 22 analysed tissues, genes under putative selection were significantly enriched for those overexpressed in adipose tissue, blood, lung, testis and uterus. Our results further suggest that *cis*-eQTL are themselves selection targets; for most tissues, we found a positive correlation between allele frequency differences and *cis*-eQTL effect size, suggesting that positive selection acts directly on regulatory variants.

**Conclusions:**

By combining selection signatures with information on gene expression and QTL, we were able to reveal compelling candidate selection targets that did not stand out from selection signature results alone (e.g. *GIMAP8* for tick resistance and *NDUFS3* for heat adaptation). Insights from this study will help to inform breeding and maintain diversity of locally adapted, and hence important, breeds.

**Supplementary Information:**

The online version contains supplementary material available at 10.1186/s12864-024-10852-8.

## Background

 Indigenous African cattle display a fascinating range of phenotypes, due to their complex admixture history and a combination of artificial selection and adaptation to the challenging environments of the African continent [1–4].

Originating from the common ancestor *Bos primigenius* (aurochs), the two *Bos taurus* subspecies, *Bos taurus taurus* (taurine cattle) and *Bos taurus indicus* (indicine cattle), migrated from their respective domestication centres at various times into the African continent via human migration and trade. In the most likely scenario, taurine cattle entered Africa via the Middle-East around 7000 − 4550 BC, and then evolved into an African taurine subspecies and dispersed further across the continent [5, 6]. An alternative scenario is that of a third domestication event from aurochs in North Africa leading to African taurine cattle [7], which has received support from several recent studies [8–10]. Domesticated indicine cattle from the Indus valley in South Asia [11] later entered Africa via the Horn of Africa in a series of migration events, starting around 700 AD [12, 13]. The two sub-species hybridised once in contact, leading to variable proportions of taurine and indicine ancestries that are detectable in modern African cattle [1, 2, 14].

The extensive genetic diversity and variation in genetic backgrounds provided the opportunity for adaptation in African cattle to a variety of environmental challenges (reviewed in Ref. [15]). Breed differentiation has resulted in more than a hundred distinct breeds [4] that can generally be grouped into breeds with high taurine (African taurine) or high indicine (“zebu” cattle) ancestries [1]. The African taurine group is generally characterised by smaller body size in comparison to zebu cattle [16] and they are also generally less susceptible to trypanosomes [17–19]. The main characteristics of zebu cattle are a large body size, a dorsal hump and better adaptation to arid climates and droughts [4].

Improved understanding of the molecular mechanisms behind the phenotypic diversity of indigenous African cattle may impact the productivity of African small-holder farming systems, and more generally, breeding decisions for livestock raised in extensive and challenging environments [20]. In the ‘State of the climate in Africa 2019’ report by the World Meteorological Organization, it was stated that Africa will be greatly affected by climate change, putting increasing strain its inhabitants and the crops and livestock on which they depend [21], possibly increasing the importance of locally adapted varieties and breeds.

Acknowledging the relevance of indigenous African cattle, much research now focuses on revealing the genetic basis of adaptive traits. For example, Wragg et al. [22] detected a resistance locus for East Coast fever (ECF) on *Bos taurus* autosome (BTA) 15 through linkage analysis. ECF is a tick-born cattle disease caused by the *Theileria parva* parasite and is among the biggest natural killers of cattle in East Africa. Other studies (reviewed in Ref. [15]) have analysed selection signatures in various local populations and breeds and identified potentially interesting regions relevant for adaptation to diverse environmental challenges (for example, in Ref. [23–26]). These studies used standard population genetics statistics, such as the integrated haplotype score (iHS), runs of homozygosity (ROH) and fixation index (*F*_*ST*_) to determine regions under selection and linked them to traits such as adaptation (e.g. trypanotolerance) and production by functional evaluation of genes located in these regions. However, determining underlying functional mechanisms of adaptation and predicting the trait associations remain major challenges in selection signature studies. To get a more comprehensive insight into trait-associations of selection, it has been suggested to link selection signatures to gene expression data [27]. Using different approaches of combining selection signatures with gene expression data or eQTL, studies have revealed potentially interesting candidate mechanisms for adaptation in humans and chickens [27–30]. For sufficient power of eQTL detection, gene expression studies require large sample sizes, and are thus costly. The cattle Genotype-Tissue-Expression atlas (CattleGTEx) is a comprehensive large-scale public database with valuable information on tissue-wise expression levels, expression quantitative-trait loci (eQTL) and transcriptome-wide association [31]. Data from the CattleGTEx project has been previously used to inform trait-associations of selection signatures in Chinese Holstein cattle [32] and American beef cattle [33].

In this study, we used whole genome sequence data from 65 samples to identify signatures of recent and incomplete sweeps in eight indigenous African cattle breeds (Ankole, Baoule, Boran, Djakkore, N’Dama, Kenana, Ogaden and Gobra). To analyse trait-associations of these selection signatures, we linked the genetic information of selection signatures to gene expression data from 22 tissues, making use of the cattle GTEx database [31]. To our knowledge, this is the first time this approach has been applied to cattle which are exposed to both artificial and natural selection pressures (i.e. indigenous populations). The dispersal of indigenous African cattle across diverse environments increases the power of such an approach to map genes driving adaptation. By investigating the trait-association of selection signatures, we hope to gain insights into which pressures predominantly shape the genetic make-up of extensively kept livestock. Specifically, the aims of this study were (a) to identify within-breed selection signatures in indigenous African cattle breeds, (b) to identify functional candidate genes for adaptation to environmental factors by connecting selection signatures with multi-tissue gene expression data and (c) to determine if expression quantitative trait loci (eQTL) show evidence of being under selection.

## Materials and methods

### Processing of whole-genome sequence data and cattle samples

In this study, we used publicly available Illumina whole-genome sequence data of global cattle breeds, for which the assembly and processing were previously described [34, 35]. The data set was filtered for at least two samples per breed and a mean sequencing depth of ≥ 8x. We further filtered for biallelic SNP variants with a call rate ≥ 95% and genotyping quality (QG) > 20. Samples with a call rate < 75% were excluded. The flag “--relatedness2” in vcftools [36] based on the method of Manichaikul et al. [37] was used to determine relationship coefficients between pairs of samples. If the relationship coefficient was > 0.177 (1st-degree relationship) between a pair of samples of the same breed, one sample was removed. Of the quality-controlled data set, we selected all African cattle breeds with at least 7 samples, resulting in 119 samples from 8 breeds (Ankole, Baoule, Boran, Djakkore, Kenana, N’Dama, Ogaden, Gobra). Boran and N’Dama cattle were overrepresented in the data set and to avoid any bias introduced by unbalanced sample sizes, we created a balanced data set (*n* = 65) in which we reduced N’Dama and Boran samples to 10 samples each using the ‘sampleCore’ function (size = 10, mode = ‘fast’) from the R package ‘corehunter’ [38]. Information on samples and accession codes are specified in Table 1. While sample sizes are low, whole genome sequence data provides the most informative marker density for selection signature statistics (including iHS), and thus is recommended for such studies, even at the cost of small sample size [39].

**Table 1:** Summary of African cattle samples analysed

Breed	Samples	Project Accession	Related publications
Ankole	7	PRJNA312138; PRJEB39282	
Baoule	7	PRJEB39924	[34]
Boran	10	PRJEB39210; PRJNA312138	[22, 40]
Djakkore	7	PRJEB39924	[34]
Kenana	9	PRJNA312138	[40]
N’Dama	10	PRJNA312138; PRJEB39353; PRJEB36894; PRJNA853448	[40, 41]
Ogaden	8	PRJNA312138	[40]
Gobra	7	PRJEB39924	

### Genomic population structure

Admixture and principal component analysis (PCA) were performed on a pruned dataset of markers (4,902,838) to reduce linkage disequilibrium (LD) between variants. Pruning was performed in Plink v1.9 [42, 43] with default settings (‘--indep 50 5 2’). The genomic structure was then analysed using PCA in Plink v1.9. Admixture software [44] was used for ancestry estimation, where the best number of clusters (K) was determined by comparing 5-fold cross-validation errors for *K* = 2,…,10. To characterise LD for each breed, we calculated the squared inter-variant allele count correlations (r^2^) for each pair of variants within 1 Mb of each other using Plink v.1.9. The mean correlation r^2^ across all variant pairs was calculated to generate the genome-wide LD level for each breed.

### Within-breed selection signatures

Within-breed selection signatures for all eight African cattle breeds were identified using the integrated haplotype score (iHS) statistic, which measures the extended haplotype homozygosity (EHH) in the genome as an indicator of recent and incomplete selective sweeps [45]. The iHS statistic is based on the integrated EHH (iHHi), which is the integral of the observed decay of EHH away from a specified core allele i until the EHH reaches a specified cut-off. Phased genotypes for SNPs across all breeds were generated using Beagle version 4.173 [46] (the phasing in Beagle was performed for Ne = 1,000 and without specifying a reference population). The ‘IMPUTE’ command in vcftools was then used to prepare input files for each breed. The software ‘hapbin’ [47] was used to calculate the variant-wise iHS statistic per breed, specifying that the iHH should be calculated up to the point at which EHH drops below 0.05 (--cutoff 0.05) for SNPs with a minor allele frequency > 0.01. The standardized iHS was calculated as in Voight et al. [45].

To determine within-breed selection signatures, we used a two-step approach: first, ‘peaks’ were identified for the SNP-wise iHS statistic (z-score). The peak calling was performed using an in-house R script [34], which screens for regions in which z-scores reach a defined maximum and then fall back below a certain minimum on either side. The maximum z-score was set to 4 and the minimum to 3.5, according to Dutta et al. [34]. Second, SNP-wise z-scores were determined for the top 0.01% for each breed (similar to Ref. [48]). The breed-specific minimum 0.01% z-score was then applied as further filter, i.e. the highest z-score of a peak had to exceed the minimum top 0.01% z-score to be considered as a selection signature. Selection signatures for each breed were mapped to the Ensembl ARS-UCD1.2 build version 96 and scanned for overlaps to identify candidate genes under selection. To identify functional gene groupings among these candidate genes under selection, we used the STRING database v12.0 [49]. Ensembl IDs of potential candidate genes under selection were uploaded to the database and default settings (organisms: *Bos taurus*, network type: full STRING network, minimum required interaction score: medium confidence 0.4, FDR stringency: medium 5%) were used to create networks. Functional enrichments in the resulting networks were reported.

To determine selection signatures that were (a) shared between breeds (“common signatures”) or that were (b) exclusive for a single breed (“exclusive signatures”), intersections between selection signatures for all breeds were identified using the option ‘multiinter’ in bedtools [50]. Intersections between within-breed selection signatures that were within 50 kb of each other, were grouped into ‘regions’ (*Reg*). Regions were again mapped to the Ensembl ARS-UCD1.2 annotation to identify candidate genes under selection.

### Tissue-specificity of candidate genes under selection

Gene expression data from CattleGTEx [31] was used to determine tissue-specific expression of genes within selection signatures. Of 8,653 RNA samples (Supplementary Table 1 in Ref. [31]), we retained 4,718 high-quality samples (using quality parameters described in Ref. [31]) comprising 22 distinct tissues (tissues with at least 40 samples: muscle, blood, liver, uterus, macrophage, embryo, rumen, mammary, adipose, ovary, pituitary, monocytes, hypothalamus, jejunum, lung, lymph node, oviduct, leukocyte, testis, ileum, skin fibroblast, salivary gland). Using the ‘limma’ package in R [51], we identified differentially expressed genes for the target tissues. This involved comparing expression for 27,607 genes between one target tissue and all others using the following pipeline: ‘lmFit’ to fit a linear model for each gene given a series of arrays, ‘contrasts.fit’ to compute estimated coefficients and standard errors for contrasts, then ‘eBayes’ to compute t-statistics, F-statistics, and log-odds of differential expression by empirical Bayes moderation of the standard errors towards a common value and finally ‘topTable’ to extract a table of all genes (no filter for top genes was applied) from the linear model fit, including FDR-correction for p-values. To determine the top over-expressed genes in a target tissue, we first filtered for log(Fold Change, FC) > 0 to select genes that are up-regulated and used the top 5% t-values (according to Ref. [31]) to select significantly over-expressed genes.

To test for enrichment of genes linked to selection signatures within genes with significant over-expression in each target tissue, we performed a Chi-squared test $\:\left({\chi\:}^{2}\right)$; we calculated the overlaps between over-expressed genes and genes that were located in selection signatures for at least one breed using the Ensembl ID identifier and compared it to overlaps between genes located in selection signatures for at least one breed and all background genes (genes that are not significantly over-expressed).

### Identifying adaptive eQTL

Information on the genomic location of significant *cis*-eQTL was available for the 22 distinct tissues (tissues with at least 40 individuals) from the CattleGTEx project [31]. In their study, most genes with significant *cis*-eQTL (eGenes) were identified in blood (10,157) and fewest genes in ileum (172 genes). For this study, the *cis*-eQTL data was not filtered for breeds (taurine, indicine, hybrids), because the limited sample size for some of the breeds decreased the power to detect breedspecific *cis*-eQTL and the study of Liu et al. [31] showed that the majority of eQTL were conserved across breeds. To determine the co-localisation between *cis*-eQTL and selection signatures, we mapped the position of variants belonging to the top 0.01% z-scores of the iHS statistic for each breed against the position of significant *cis*-eQTL for each tissue.

To test whether selection signatures overlapped with tissue-specific *cis*-eQTL more than expected by chance, we performed a permutation test using the R package ‘regioneR’ [52]. We mapped the overlap between all identified selection signatures (iHS peaks with variants belonging to the top 0.01% z-scores) and the position of *cis*-eQTL in each tissue, and compared it to 10,000 random sets generated by circular randomization (‘circularRandomRegions’ function). Circular randomization, where the randomization process maintains the order and distance of the regions while changing their position in the chromosome, was chosen as the randomization strategy in order to preserve the chromosomal structure of cattle.

To determine whether variants under selection affect gene expression (adaptive eQTL), we calculated the Spearman correlation between across-breed allele frequency differentiation and the effect size (slope) of *cis*-eQTL. For this analysis, we were interested in general allele frequency differences across all breeds and therefore focused on a cross-breed statistic; we used Plink v1.9 (specifier --fst --within) to calculate allele frequency differentiation across all cattle populations (global *F*_*ST*_) for each variant. Then we used “ggscatter” from the R package ‘ggpubr’ to visualise and calculate the Spearman correlations between the global *F*_*ST*_ and *cis*-eQTL slope for overlapping variants. Extreme variants, those for which a visual inspection indicated the *cis*-eQTL effect size was substantially higher (greater than 390 times) than the average of all other values for a tissue, were excluded for that tissue; this affected ‘Embryo’ (1 variant) and ‘Oviduct’ (2 variants). Similar to Quiver et al. [28], the difference between the *cis*-eQTL slope for adaptive variants (high global *F*_*ST*_, defined as the top 1% variants for this statistic) and non-adaptive variants was tested using a Wilcoxon test (implemented in the ‘rstatix’ R package).

### Trait-association of selection signatures

The Cattle QTL database (CattleQTLdb; https://www.animalgenome.org/cgi-bin/QTLdb/BT/index; Ref. [50]), which comprised 196,904 QTL/associations (Release 52, Dec 23, 2023), was used to map selection signatures to QTL for various traits. Specifically, the coordinates of selection signatures were compared to that of the CattleQTLdb using ‘intersect’ in bedtools [50].

## Results

### Population structure

The genetic PCA revealed a clustering of N’Dama and Baoule, with negative scores for PC1, with the remaining breeds having positive scores for PC1 (see Additional file 1; Figure S1). For the Admixture analysis, the lowest cross-validation error was observed for *K* = 2. For *K* = 2, breeds were assigned to a light green cluster (which can be viewed as a proxy for African taurine background) and a light orange cluster (proxy for ancestries with a non-taurine background) (see Additional file 1; Figure S2). With increasing *K*, breeds showed higher diversification, i.e. Boran separated from other non-taurine breeds from *K = 4*, and Ankole separated at K = 5.

### Comparative analysis of within-breed selection signatures

We identified peaks of the iHS statistic (z-scores) for each breed and considered peaks with at least one variant that exceeded the minimum top 0.01% z-score as selection signatures. Number and average length of selection signatures differed between breeds (Table 2), with Kenana, Djakkore and Boran having the highest number, but on average shorter, selection signatures and Baoule and N’Dama having the lowest number, but on average considerably longer, selection signatures. This may be a consequence of higher linkage disequilibrium in Baoule and N’Dama, as suggested by genome-wide average *r*^*2*^ values. The highest z-scores were detected for Boran, followed by Gobra.

**Table 2:** Summary statistics for within-breed selection signatures

Breed	min z-score top 0.01%^$^	*n*	Length (bp)	max z-score	mean (median) *r*^2^
Ankole	4.71	229	5717.58	5.63	0.74 (1)
Baoule	5.99	95	9562.05	6.99	0.81 (1)
Boran	7.49	435	3331.46	9.22	0.71 (0.78)
Djakkore	5.28	470	2008.72	6.18	0.71 (0.78)
Kenana	4.92	476	3211.16	5.81	0.70 (0.73)
N’Dama	5.28	132	10165.92	6.03	0.79 (1)
Ogaden	4.80	426	2732.01	5.78	0.71 (0.77)
Gobra	6.38	365	3629.89	7.46	0.71 (0.77)

^$^used as cutoff for peaks in the two-step filter for selection signatures

n: number of selection signatures

Length: Average length of selection signatures in bp

Max z-score peak: Highest z-score for selection signature

*r*^2^: Genome-wide mean (median) squared inter-variant allele count correlations for each pair of variants within 1Mb of each other

### Selection signatures within breeds

Ankole: Among the signatures with the highest z-scores, all comprised uncharacterized proteins except one signature, which contained the gene *TSPEAR* (BTA1) (see Additional file 2; Table S1). The longest selection signature was located on BTA29 (containing two genes: one encoding a putative ankyrin repeat domain-containing protein and the other encoding an uncharacterized protein belonging to the tropomyosin family).

Baoule: The signature with the highest z-scores was an extended cluster located on BTA9 84-88Mbp (three genes; including *ULBP17*), while the longest region was located on BTA26 (*DOCK1*, *NPS*).

Boran: The region with the highest z-scores was located on BTA10 (25 Mb). The longest region was detected on BTA12, followed by a region on BTA28 containing three genes, two of which are olfactory receptor genes (*OR5L2*,* OR5AS1*).

Kenana: The selection signature with the highest z-scores involved two neighbouring regions around 72 Mb on BTA12. The longest selection signature was located on BTA17 (three genes: *MLXIP*,* BCL7A*,* WDR66*).

Ogaden: The highest z-score was observed for a selection signature on BTA20 (0.1 Mb) and the longest signatures were found on BTA29 and BTA7, which included 9 genes (*MADCAM1*, *TPGS1*, *CDC34*, *GZMM*, *BSG*, *HCN2*, *POLRMT*, *FGF22*, *RNF126*).

For Djakkore, N’Dama and Gobra the highest z-score and longest selection signatures did not contain any protein coding genes.

We used STRING to identify functional gene groupings among genes located within selection signatures for each breed (see Additional file 3; Table S2). Genes located within selection signatures were significantly enriched for terms related to the adaptive and innate immune response, i.e. immunoglobulin V-related STRING clusters were enriched for Ankole, Boran, Djakkore, Kenana and Ogaden (mostly involving genes coding for Ig-like domain-containing proteins). In Baoule, multiple immune system-related Gene Ontology (GO) terms and MHC-related STRING clusters were enriched. Phospholipase A2 genes, which were located in selection signatures in Kenana (*PLA2G2D1*,* PLA2G5*), led to the enrichment of phospholipase A2-related InterPro (catalogue of protein families) terms.

### Common and exclusive selection signature regions

**Fig. 1:**
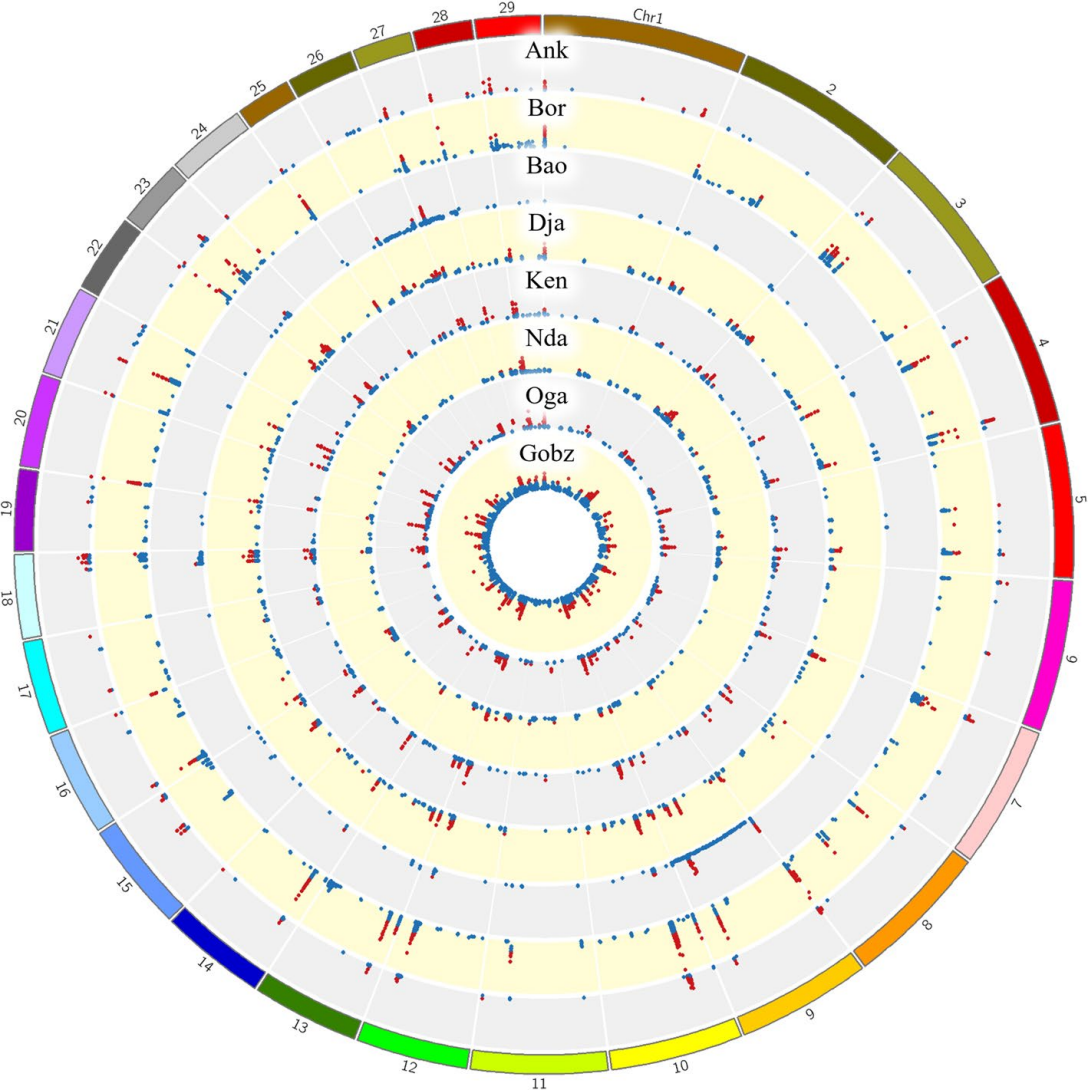
Genomic location of within-breed selection signatures. Selection signatures were determined based on a two-step approach: first, ‘peaks’ were identified for the SNP-wise iHS statistic (z-score) by screening for regions in which z-scores reached a maximum of 4 and then fell below 3.5 on either side. Each point in the Circos plot represents the maximum z-score of one such peak. Second, z-scores were screened for the top 0.01% for each breed and the minimum z-score for those top 0.01% per breed was used as further filter; the maximum z-score of a peak had to exceed the minimum top 0.01% z-score for that breed to be considered as selection signature (red points). Breed abbreviations: ANK, Ankole; BAO, Baoule; BOR, Boran; DJA, Djakkore; GOBZ, Gobra; KEN, Kenana; NDA, N’dama; OGA, Ogaden

**Fig. 2:**
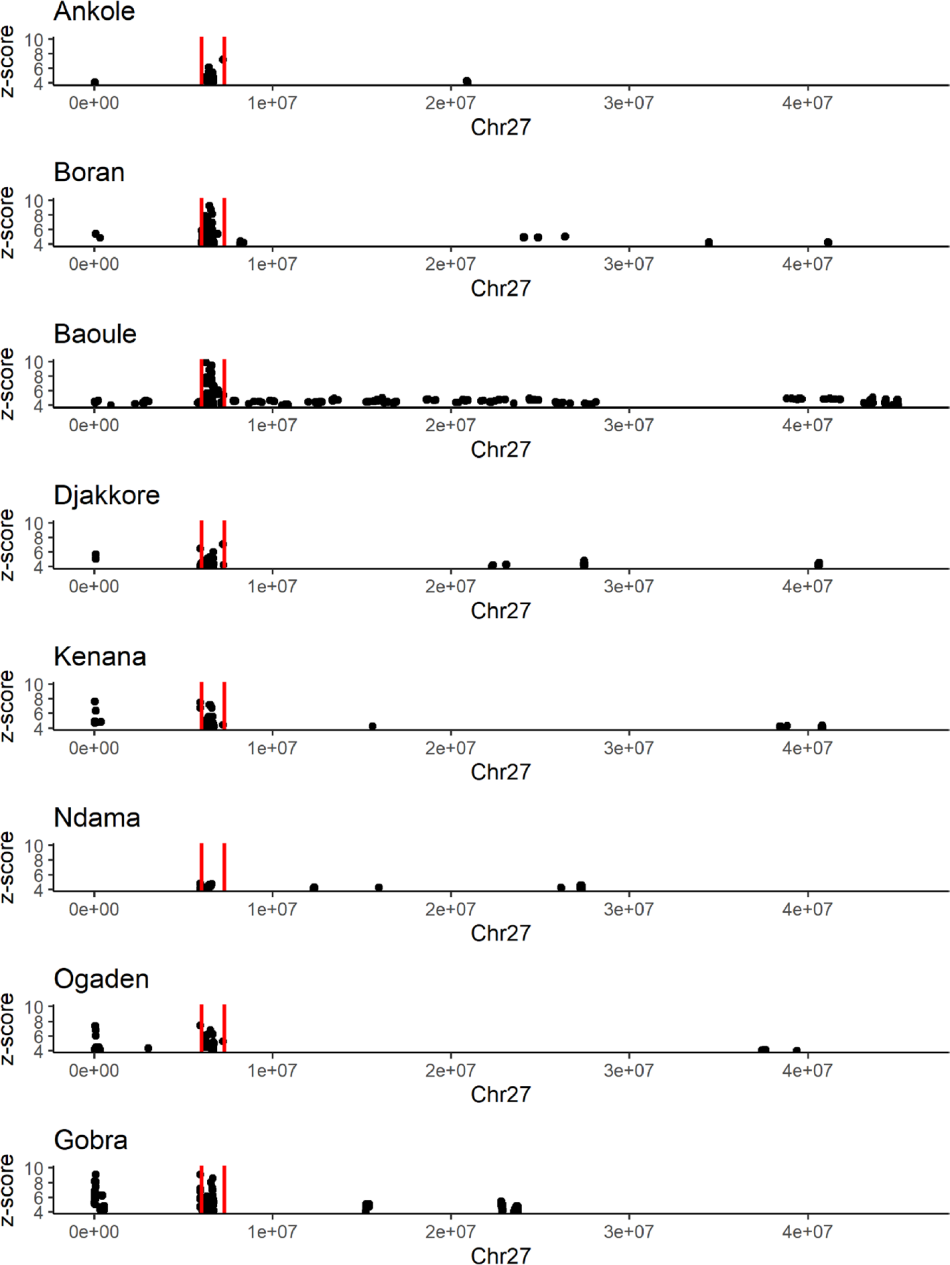
Location of selection signatures on BTA27 overlaps with DEFB genes. The iHS (z-scores) were plotted for selection signatures in relation to the genomic locations of 29 bovine DEFB genes (according to Ref. [53] ) , the range of which is indicated by the two red vertical lines

 Some selection signatures showed overlaps between breeds while others showed breed-specificity (Fig. 1). To determine selection signatures that were shared between breeds (“common signatures”) or that were exclusive for a single breed (“exclusive signatures”), we calculated intersections and grouped intersections within 50 kb of each other into ‘regions’ (*Reg*). Following this procedure, we detected 322 regions of intersections (see Additional file 4; Table S3). There are three regions for which all eight breeds had a selection signature: Reg_112 (BTA9; no genes), Reg_160 (BTA12; including a single uncharacterised protein encoding gene) and Reg_176 (BTA14; including two genes encoding protein kinase domain-containing proteins). On BTA2, we identified a cluster of common selection signatures for Djakkore, Kenana, Ogaden and Gobra containing several phospholipase A2-associated genes (*PLA2G2D1*,* PLA2G5*,* PLA2G2A*,* ENSBTAG00000048919*,* PLA2G2A).* On BTA27, all breeds except N’Dama shared a common selection signature comprising seven genes (Reg_303), three of which are beta defensins (*DEFB10*,* DEFB7*,* DEFB1*). This region overlaps a large cluster of beta defensin genes described in Ref [53]. (Fig. 2).

The longest selection signature region overlapping candidate genes was located on BTA12 (Reg_158), which was Boran-specific (encoding an uncharacterized protein). The second, third and fourth longest regions (Reg_135, Reg_137, Reg_133, including 9, 11 and 11 genes respectively) were located on BTA10 and Reg_135 and Reg_137 were detected for all breeds except Baoule. The STRING enrichment for these genes was associated with terms of the immune response. Other long regions were detected on BTA5 (Reg_71; *WC1.3*,* WC1*, gene encoding PCI domain-containing protein; Djakkore, Kenana, Ndama, Ogaden and Gobra), BTA1 (Reg_3; including a single gene encoding an uncharacterised protein; found in all breeds except N’Dama and Baoule) and BTA26 (Reg_296; *DOCK1*,* NPS;* Baoule).

### Tissue-specificity of genes under selection and adaptive eQTL

A total of 350 genes overlapped with the 322 regions of intersections between breed-specific selection signatures and were considered as candidate genes under selection. To test for enrichment of this set of candidate genes under selection in the set of genes that are over-expressed in specific tissues, we performed a $\:{\chi\:}^{2}$ test. Genes under selection were significantly enriched in genes over-expressed in adipose, blood, lung, testis and uterus tissue (Table 3). According to STRING, genes that overlapped between selection signatures and over-expression in blood (63 genes) were enriched for the GO Molecular functions ‘Peptide antigen binding’ and ‘Scavenger receptor activity’. These included multiple *white collar* (*WC*) genes, belonging to the scavenger receptor cysteine-rich (SRCR) superfamily. For genes that overlapped between selection signatures and over-expression in adipose tissue (7 genes), testis (14 genes) and uterus (7 genes), no significant enrichment was detected. For the tissues with significant enrichment of selection signature genes (Table 3), the genes with the highest Fold Change (FC) that overlapped with genes within selection signatures were the following: for adipose tissue, *PCOLCE2* (Procollagen C-endopeptidase enhancer 2; selection signatures only in Ankole); for blood, *GIMAP8* (GTPase, IMAP family member 8; selection signatures only in Boran), for lung, *LOC510193* (selection signature only in N’Dama); for testis, ENSBTAG00000051666 (selection signature in Ogaden and Gobra) and for uterus, *FNDC1* (Fibronectin type III domain containing 1; selection signatures only in Ogaden).

We then tested if selection signatures and *cis*-eQTL overlapped more than expected by chance using a permutation test and compared the overlap with 10,000 random sets generated by circular randomization. The overlap was higher for selection signatures compared to random data sets and for 11 out of the 22 tissues this enrichment was significant, indicating that *cis*-eQTL are more enriched in selection signature loci than other regions of the genome (Table 3). Adipose, blood and uterus tissue showed significant results for both analyses, enrichment of genes under selection in genes over-expressed in the tissues and enrichment of overlap between the position of selection signatures and *cis-*eQTL.

**Table 3:** Overlap between gene expression and ***cis***-eQTL with selection signatures. To test for enrichment of genes under selection in genes over-expressed in distinctive tissues (a), a Chi-squared test was performed between genes that overlapped with a selection signature in at least one breed (*n* = 350) and the top 5% of genes that were over-expressed in 22 distinctive tissues. To test for enrichment of selection signatures within *cis-*eQTL (b), a permutation test was performed

Tissue	a) Enrichment of selection candidate genes in tissue-specific over-expressed genes (Chi-squared test)	b) Enrichment of selection signatures in cis-eQTL (permutation test)	*p*-value
	*p*-value	z-score	
**Adipose**	1.20E-03*	4.39	6.00E-04*
**Blood**	1.72E-73*	5.76	1.00E-04*
Embryo	1.32E-01	2.63	1.34E-02
Hypothalamus	1.45E-01	3.57	4.20E-03
Ileum	3.87E-03	2.07	5.97E-02
Jejunum	3.43E-02	7.68	1.00E-04*
Leukocyte	2.38E-02	6.73	1.00E-04*
Liver	1.48E-01	3.43	2.70E-03
Lung	4.73E-04*	2.77	8.90E-03
Lymph node	3.56E-03	5.08	2.00E-04*
Macrophage	2.70E-02	2.41	1.86E-02
Mammary	3.08E-03	5.05	1.00E-04*
Monocytes	5.25E-03	6.17	1.00E-04*
Muscle	6.73E-01	4.18	1.00E-03*
Ovary	6.13E-03	5.27	1.00E-04*
Oviduct	1.57E-02	3.26	9.70E-03
Pituitary	7.75E-03	4.22	2.60E-03
Rumen	1.61E-01	6.69	1.00E-04*
Salivary gland	6.39E-03	2.58	2.45E-02
Skin fibroblast	4.06E-03	-0.11	6.09E-01
Testis	3.17E-04*	1.71	7.75E-02
**Uterus**	1.56E-03*	9.1	1.00E-04*

*Significantly enriched for Bonferroni-corrected *p*-value (0.05/22)

Tissues in bold had a significant result in both analyses.

**Fig. 3:**
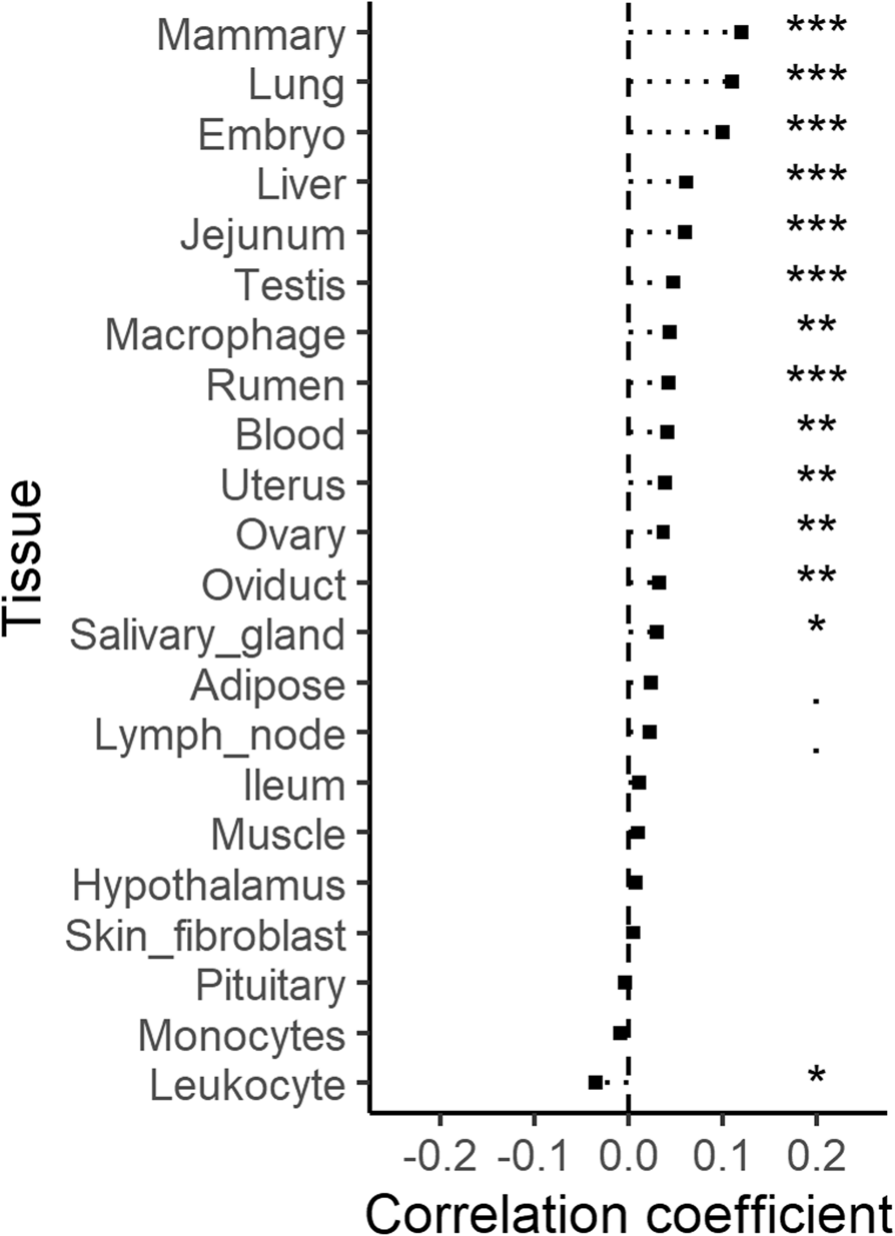
Spearman correlation coefficients between *F*_*ST*_ and cis-eQTL slope (effect size) across all markers for each tissue. The significance of the regression is indicated by 0–0.001 = ‘***’; 0.001–0.01 = ‘**’; 0.01–0.05 = ‘*’ and 0.05–0.1= ‘.‘

 To address the question of whether *cis*-eQTL are directly under selection (as opposed to coinciding with genes under selection), we calculated the correlation between allele frequency differentiation and the effect size (slope) of *cis*-eQTL (Fig. 3; Additional file 5, Figure S3). We observed the highest positive correlation for ‘Mammary’ tissue, followed by ‘Lung’, both of which were also highly significant. Out of the 22 tissues tested, the effect size of *cis*-eQTL of 14 tissues was significantly positively correlated with the global *F*_*ST*_ values.

We also determined the difference between the *cis*-eQTL slopes for “adaptive” (top 1% for global *F*_*ST*_) and “non-adaptive” (all other) variants (see Additional file 6; Table S4). For 11 tissues, adaptive variants had higher effect sizes than non-adaptive variants. The most significant result and the greatest difference between adaptative and non-adaptive variants was detected for lung tissue, with the variant affecting expression of the gene *FAF1* having the greatest slope. Furthermore, we found significant associations between allele frequency differentiation and *cis*-eQTL effect size for tissues of female reproductive organs, i.e. ovary and oviduct showed a significant positive correlation coefficient and a highly significant difference between adaptive and non-adaptive variants, uterus showed a highly significant positive correlation coefficient.

### Trait-association of selection signatures

**Fig. 4:**
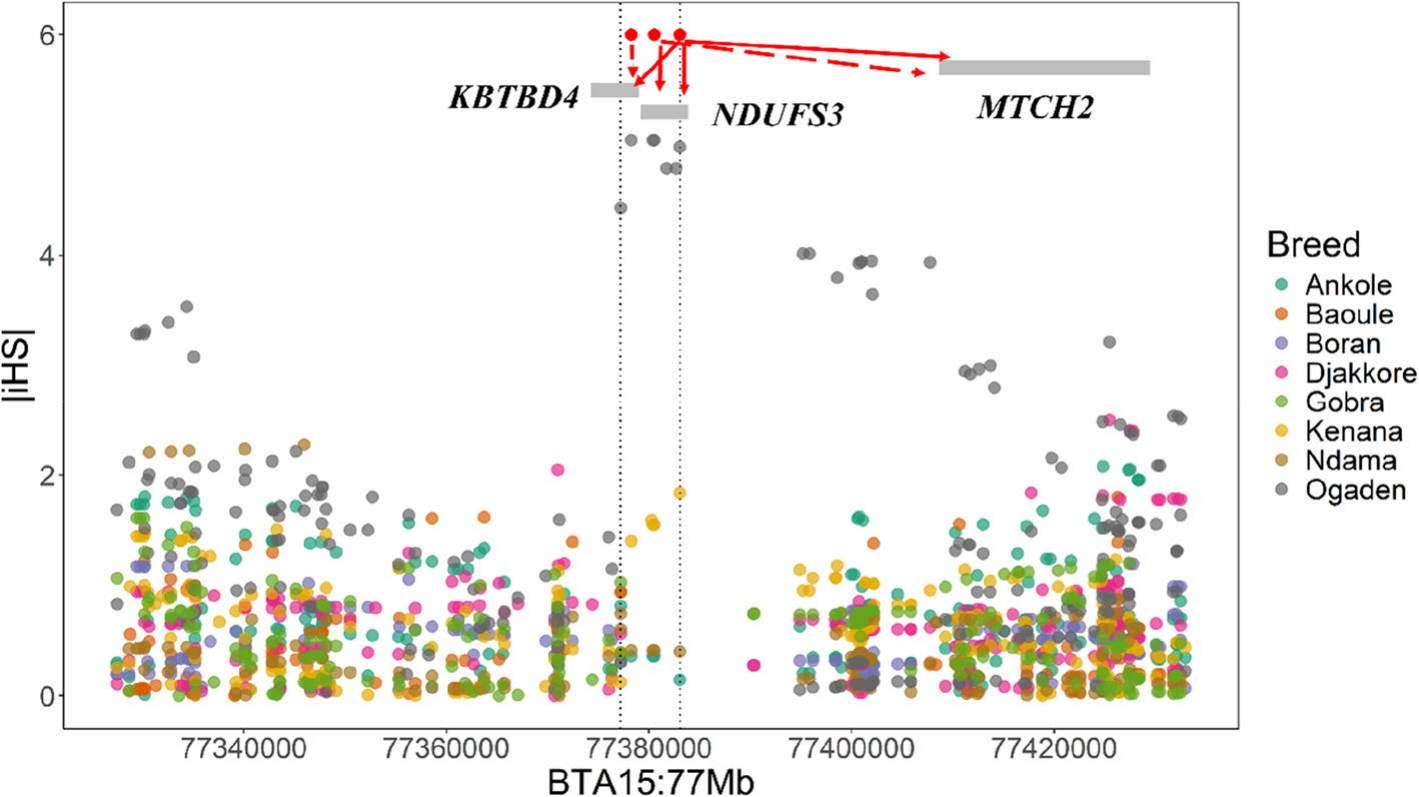
Overlaps between selection signatures and cis-eQTL on BTA15:77.3Mb. The figure shows the z-scores for the respective breeds with a clear selection signature for Ogaden, red points indicate the location of cis-eQTL and the arrows indicate the genes with which the cis-eQTL is associated

 We mapped positions of *cis*-eQTL to selection signatures to identify possible adaptive eQTL and their targets. The genomic positions of significant *cis*-eQTL across 22 tissues reported in Liu et al. [31] were compared to the positions of variants within the top 0.01% of z-score statistics for the iHS analysis of each breed. We identified 30 overlaps between significant *cis*-eQTL and top z-scores, on BTA1, 2, 4, 5, 6, 7, 8, 9, 10, 15, 16, 17, 18 and 23, with a cluster of overlaps at BTA15:77.3 Mb (Table 4; Fig. 4).

**Table 4:** Co-localisation between ***cis***-eQTL and selection signatures. The location of significant *cis*-eQTL for 22 tissues from the cattle GTEx database was mapped to the location of variants belonging to the top 0.01% z-score statistic for each African cattle breed

eVariant^*^	Chr	Pos	Tissue	eGene^$^	Breed	z-score
1_555602_G_A	1	555,602	Embryo	*KCNE1*	Boran	9.67
2_132628463_C_T	2	132,628,463	Lymph node	*HTR6*	Kenana	4.98
4_105601393_G_T	4	105,601,393	Uterus	*ENSBTAG00000050211*	Gobra	6.69
5_69741576_C_A	5	69,741,576	Blood	*POLR3B*	Kenana	4.94
6_69791614_A_G	6	69,791,614	Blood	*PDGFRA*	Ankole	5.03
7_43171756_G_A	7	43,171,756	Embryo	*MGC137030*	Ogaden	4.96
7_43198873_C_A	7	43,198,873	Uterus	*SH3BP5L*	Ogaden	4.96
7_43249491_T_G	7	43,249,491	Uterus	*SH3BP5L*	Ogaden	4.96
7_43249491_T_G	7	43,249,491	Macrophage	*MADCAM1*	Ogaden	4.96
7_43446904_C_A	7	43,446,904	Macrophage	*CNN2*	Ogaden	4.88
8_23127915_T_A	8	23,127,915	Adipose	*HACD4*	Ogaden	5.03
8_23149470_C_A	8	23,149,470	Blood	*ENSBTAG00000052820*	Ogaden	4.90
8_23153052_C_T	8	23,153,052	Blood	*ENSBTAG00000053413*	Ogaden	4.90
8_23254079_T_C	8	23,254,079	Liver	*IF1DA6*	Kenana	5.06
9_86988594_T_C	9	86,988,594	Macrophage	*PLEKHG1*	Baoule	7.39
10_22912865_T_C	10	22,912,865	Blood	*ENSBTAG00000054091*	Ogaden	5.58
15_46370164_T_C	15	46,370,164	Lymph node	*ENSBTAG00000049294*	Ogaden	5.49
15_46370180_T_C	15	46,370,180	Liver	*DCHS1*	Ogaden	4.97
15_77378244_C_T	15	77,378,244	Rumen	*KBTBD4*	Ogaden	5.05
15_77380548_C_A	15	77,380,548	Muscle	*NDUFS3*	Ogaden	5.05
15_77380548_C_A	15	77,380,548	Jejunum	*MTCH2*	Ogaden	5.05
15_77383059_C_T	15	77,383,059	Liver	*KBTBD4*	Ogaden	4.98
15_77383059_C_T	15	77,383,059	Muscle	*NDUFS3*	Ogaden	4.98
15_77383059_C_T	15	77,383,059	Blood	*MTCH2*	Ogaden	4.98
15_77383059_C_T	15	77,383,059	Lymph node	*MTCH2*	Ogaden	4.98
16_1969224_T_G	16	1,969,224	Uterus	*ETNK2*	Kenana	5.02
16_27351343_G_A	16	27,351,343	Muscle	*NVL*	Ndama	5.37
17_26422_G_T	17	26,422	Monocytes	*TMEM192*	Gobra	10.37
18_63477247_T_C	18	63,477,247	Blood	*ENSBTAG00000039086*	Ogaden	4.82
23_28909864_C_T	23	28,909,864	Liver	*POLR1H*	Kenana	5.04

^*^eVariant: ID of significant *cis*-eQTL

^$^eGene: target gene of significant *cis*-eQTL

We also used information from CattleQTLdb to identify overlaps between breed-wise selection signatures and QTL. The number of overlaps and the predominantly enriched trait classes varied between breeds; we found the most overlaps for Kenana selection signatures and the least for Baoule (see Additional file 7; Table S5, Additional file 8; Figure S4). For all breeds except Baoule and N’Dama, there was overlap between a selection signature on BTA1 and a QTL for “Subcutaneous fat thickness” (Table S5; QTL ID = 281728). Selection signatures in Boran, Ogaden and Djakkore showed the greatest number of overlaps with traits associated with “meat and carcass”, while in Baoule, Ankole and Gobra, selection signatures showed the same number of overlaps with traits associated with “milk” and “meat and carcass”. In N’Dama, selection signatures overlapped primarily with the “reproduction” trait class. In Kenana, the selection signatures overlapped primarily with the trait class “exterior”.

## Discussion

Characterising the genetic basis of environmental adaptation and artificial selection for indigenous livestock species remains a major aim in the context of a changing climate and commercial landscape. To counteract the potential loss of genetic diversity due to targeted breeding and introduction of exotic breeds, and to maintain variation associated with important traits, it is relevant to identify functionally important areas of the genome. In this paper, we performed selection signature scans in indigenous African cattle breeds that are adapted to different environments and subject to different human-imposed selection pressures. To gain insights into the traits that are driving genomic signatures of selection, we linked these signatures of recent and incomplete sweeps to gene expression data and QTL information. As a result, some of the regions under selection we detected overlapped with previously identified candidate genes for adaptation in African cattle (reviewed in Ref. [15, 54]) (see Additional file 1; Table S5 and 6). However, by using additional information from gene expression and QTL studies, we also identified novel candidate genes, which are discussed in more detail below.

### Traits under selection

One major challenge in selection signature studies is to determine which trait(s) are driving genomic signatures. In indigenous African cattle, selective forces could be related to specific environmental factors, e.g. selection for trypanotolerance, heat and drought tolerance or tick resistance [15], or to artificial selection for performance traits, e.g. milk yield, meat quality and fertility. While numerous selection signature studies have been carried out in African cattle (for example, in Ref. [23–26]), pinpointing the causal variants and underlying genes remains challenging because large genomic regions containing multiple genes are usually identified as selective sweeps. With the use of whole-genome sequence data it is possible to narrow down candidate genes, as demonstrated in Boitard et al. [55]. However, downstream analyses (e.g. gene knockout studies) are usually required to identify the function of the gene and thus confirm the trait under selection. As an alternative approach to address this challenge, recent studies in humans and chickens have complemented selection signatures with gene expression information to draw more informed conclusions about adaptive variation [27–30]. In this study, we focussed on dissecting traits under selection in indigenous African cattle by the joint analysis of selection signatures and gene expression (cattle GTEx) and QTL (CattleQTLdb) databases.

Overall, results from the joint analysis of selection signatures, gene expression and QTL in this study suggest the immune response (e.g. resistance to diseases) as well as reproduction and production traits as important selective pressures in African cattle.

#### Immune response

Our investigation of selection signatures and the correspondence with gene expression patterns points towards a role of the immune system in the adaptation of indigenous African cattle, which is consistent with other studies (e.g. in Ref. [56]).

We found that genes located within selection signatures were significantly enriched for terms of the adaptive and innate immune response, in accordance with the known variability in susceptibility and resistance levels of indigenous African cattle to diseases and pathogens (reviewed in Ref. [54]). For example, immunoglobulin V-related STRING clusters (mostly genes coding for Ig-like domain-containing proteins) were enriched for Ankole, Boran, Djakkore, Kenana and Ogaden and multiple pathogen defence-related GO terms and MHC-related STRING clusters were enriched in Baoule. Furthermore, the genomic region on BTA2:132 Mb, where we identified a cluster of selection signatures for Djakkore, Kenana, Ogaden and Gobra, contains a cluster of phospholipase A2-associated genes (*PLA2G2D1*,* PLA2G5*,* PLA2G2A PLA2G2A*), which are involved in the innate immune response [57].

Linking genes under selection to gene expression, we were able to further suggest specific candidate genes for the immune response. Genes involved in immune response pathways have been shown to be continuously expressed in whole blood gene expression profiles in humans [58]. We detected a significant enrichment of genes located in selection signatures within genes that were overexpressed in blood (Table 3). Correspondingly, in the STRING analysis it was revealed that these overlapping genes were mainly involved in pathways of the immune response (GO Molecular functions ‘Peptide antigen binding’ and ‘Scavenger receptor activity’). The 63 overlapping genes between those in selection signatures and those over-expressed in blood included multiple *white collar* (*WC*) genes belonging to the scavenger receptor cysteine-rich (SRCR) superfamily, which are relevant for pathogen recognition. Specifically, WC1 receptor genes were located in a long selection signature for Djakkore, Kenana, Ndama, Ogaden and Gobra on BTA5 (5th longest; Reg_71; *WC1.3*, *WC1*). *WC1* receptors are exclusively expressed in gamma delta T cells and cluster in two genomic regions on BTA5 in cattle [59].

The gene *GIMAP8* had the highest fold-change (FC) of expression in blood and was also located in a selection signature identified in Boran. *GIMAP8* belongs to the GTPase family of immunity-associated proteins (GIMAPs), which are predominantly expressed in immune cells like T and B cells [60]. In a previous study on the immune response to tick infestation in cattle breeds with varying resistance levels, it was observed that B leucocytes mediate the increase in CD3^+^ T lymphocytes, which was characteristic for tick resistant breeds [61]. In a systematic review of GWAS results with other information (e.g. Variant Effect Predictor from Ensembl), *GIMAP8* was identified as major candidate gene for tick resistance in cattle [62]. The significantly higher expression of *GIMAP8* in blood (including T and B cells) compared to other tissues makes it a compelling candidate for selection for tick resistance.

#### Production and reproduction traits

We found genes under selection to be significantly enriched in genes that are over-expressed in adipose tissue, testis and uterus. The gene *PCOLCE2* (BTA1:126 Mb), which showed selection signatures in Ankole, had the highest FC in expression in adipose tissue compared to all other tissues. A study in mice and humans showed that the expression of *PCOLCE2* in adipose tissue was positively correlated with adipose mass and also had an impact on cholesterol catabolism, suggesting a novel function of the gene in adipose tissue expansion and cholesterol balance [63]. The finding that *PCOLCE2* shows significantly higher expression in adipose tissue compared to other tissues in cattle provides further evidence for the link between this gene and adipose mass. Combining this information with the selection signature in Ankole, *PCOLCE2* could be a candidate gene for selection for meat-related traits. Correspondingly, Ankole cattle are considered to be a breed with good potential for beef production and multiple genomic regions under selection have previously been associated with meat quality [26].

We further examined the potential traits under selection by comparing the identified selection signatures to positions of QTL from the CattleQTLdb database [64]. Most overlaps were found between selection signatures and production traits. Traits associated with “meat and carcass” showed the greatest number of overlaps in Boran, Ogaden and Djakkore, while traits associated with “milk” showed the same number of overlaps as “meat and carcass” in Baoule, Ankole and Gobra. The QTL trait class that overlapped with the greatest number of selection signatures in N’Dama was “reproduction”. The observation that selection signatures mostly overlapped with production traits could lead to the conclusion that selection for these traits (artificial selection) is the major driving force behind diversity in indigenous African cattle. However, it needs to be noted that the CattleQTLdb is heavily biased towards QTL associated with production traits (i.e. they substantially outnumber QTL potentially underlying natural selection such as health traits) and therefore this conclusion cannot be drawn.

Some specific candidate regions for production and reproduction traits emerged in the joint analysis of selection signatures and QTL. For example, all of the breeds except N’Dama and Baoule showed a selection signature on BTA1, which overlapped with a QTL for “Subcutaneous fat thickness” previously detected in Nellore [65]. Considering the underlying trait for the QTL and the occurrence of the selection signature only in indicine African cattle, this could suggest a contribution of that genomic region to hump size and development, which varies across indicine cattle [66]. Furthermore, all breeds except Baoule and Djakkore showed a selection signature on BTA29 that overlapped with a QTL associated with reproduction traits (interval to first oestrus after calving, age at puberty) in Australian Brahman and hybrids adapted to tropical climates [67]. Correspondingly, fertility traits have been suggested to be under selection in African cattle in various other studies (reviewed in Ref. [15]). Furthermore, in Kenana, selection signatures on BTA16 overlapped with QTL associated with udder phenotypes that have been shown to be correlated to milk performance traits in tropical cattle [68]. Kenana has been classified as among the highest milk production breeds in Sudan [69], thus this finding further supports selection for milk performance in Kenana [70].

#### Other traits under selection

When comparing the overlap between selection signatures and *cis*-eQTL identified from the Cattle GTEx data [31], we identified a cluster of overlaps between an Ogaden-specific selection signature and *cis*-eQTL on BTA15:77.3 Mb. This cluster contained *cis*-eQTL acting on the expression of the genes *KBTBD4* in rumen and liver, *NDUFS3* in muscle and *MTCH2* in jejunum, blood and lymph node. *NDUFS3* and *MTCH2* are both involved in mitochondrial energy production. Variation in *NDUFS3* was previously found to be significantly associated with seasonal hair shedding in cattle [71] and the gene was also located in a selection signature in cattle and two other *Bos* species adapted to different climates [72]. Ogaden is a dairy type cattle in tropical countries where adaptation to heat stress can be considered as a major selection factor [73]. By combining the selection signature results with *cis*-eQTL data in this study *NDUFS3* emerges as a compelling candidate gene for adaptation to heat stress and thermotolerance.

Some candidate genes with relevant functions were revealed only in the selection signature analysis; for example, in Ankole, the signatures with highest z-scores all comprised uncharacterized proteins except one signature that contained the gene *TSPEAR* (BTA1: around 1.44 Mb) (see Additional file 2; Table S1), which was also within selection signatures in Ogaden and Gobra. The *TSPEAR* gene has been shown to be a regulator of the notch signalling pathway affecting tooth and hair follicle morphogenesis [74]. This region is near (1.39 Mb) a SNP within the gene *IFNGR2*, which segregated perfectly with polledness in Holsteins [75]. In another study in cattle, a region showing evidence of differentiation between Norwegian Red-polled cattle and a related un-polled breed included *TSPEAR* [76]. Ankole cattle have distinctive long horns, while the horn phenotype is more variable in Ogaden and Gobra, suggesting this region on BTA1 including *TSPEAR* as a candidate region for horn growth and morphology.

### Are eQTL under selection?

Within the study, we addressed the question of whether selection signatures and *cis*-eQTL are co-localised, and if so, whether there is evidence that variants acting as *cis*-eQTL are directly under selection. In summary, our results suggest that eQTL are selection targets. First, we compared the overlap between selection signatures and *cis*-eQTL identified from the Cattle GTEx data [31] and found 30 overlaps between significant *cis*-eQTL and top z-scores (on BTA1, 2, 4, 5, 6, 7, 8, 9, 10, 15, 16,17, 18 and 23). Based on a permutation test we found that *cis*-eQTL were significantly more likely to be located in selection signatures (Table 3). Furthermore, for most tissues, we found a positive correlation between allele frequency differentiation and *cis*-eQTL effect size, suggesting that positive selection acts directly on regulatory variants, which is in line with other studies in pigs [77] and humans [78]. Selection on eQTL might be particularly effective for short-term adaptation, because variants can alter gene expression only for specific tissues. Studies have demonstrated that the plasticity of gene expression can be important in responses to rapid environmental changes [79].

The greatest significant difference between eQTL effect size of adaptive and non-adaptive variants (top 1% *F*_*ST*_ vs. all others) was observed for lung, with the variant affecting the gene *FAF1* having the greatest eQTL slope. This eQTL is a compelling target for association with tolerance to East Coast fever in African cattle, as a paralogue of *FAF1* was found to be highly associated with disease survival in Boran and the lung is a key organ for infestation with *Theileria parva*, the disease-causing agent [80]. Additionally, the allele frequency of the alternative allele is higher in Eastern African cattle breeds in this study, suggesting an introduction of this allele from the East, potentially as an adaptation to the disease.

## Conclusions

The phenotypic diversity of indigenous African cattle is mirrored in their genetic makeup. In this study we identified both common and breed-specific selection signatures. By combining the selection signatures with information on gene expression and QTL, we conclude that immune response, production and reproduction are targets of selection. Furthermore, the approach of combining different data types revealed compelling candidate genes for environmental adaptation that did not stand out from selection signature data alone (e.g. *GIMAP8* and *NDUFS3).*

This study also provided evidence that positive selection directly acts on regulatory variants, with emphasis on genes of the female reproduction organs and lung. The lung plays the role of a primary physiological and immunological barrier to infections and thus might be under selection from rapidly-evolving pathogens, suggesting a role for regulatory variants due to their plasticity.

While sample sizes in this study are low, we feel confident that the choice of the iHS method and the strict criteria for identifying selection signatures promoted the detection of true signals, which is why we found signatures in common with other studies and also shared between breeds in our study. Ideally, for a more comprehensive picture, a similar study should be conducted on a larger African cattle data set. In general, utilising large scale data from multiple levels (e.g. genome, transcriptome) increases the study power. However, most of the publicly available data sets are skewed towards production-intensive Western breeds and thus, unique adaptation mechanisms of native breeds might be missed. Future studies will benefit from functional data for those breeds, which will provide better insights into the genetic basis of adaptation of less commercialised livestock populations.

## Supplementary Information

Additional file 1: Figure S1. Principal component analysis of African cattle breeds. Eigenvectors for the first two principal components are plotted and the variances explained by the principal components are given in parentheses, with samples coloured by breed (abbreviations: ANK, Ankole; BAO, Baoule; BOR, Boran; DJA, Djakkore; GOBZ, Gobra; KEN, Kenana; NDA, N’dama; OGA, Ogaden). Figure S2. Admixture analysis of African cattle breeds. Proportion of genetic admixture for *K* =1, … ,5 of 65 African cattle samples. Breed abbreviations: ANK, Ankole; BAO, Baoule; BOR, Boran; DJA, Djakkore; GOBZ, Gobra; KEN, Kenana; NDA, N’dama; OGA, Ogaden. Table S5. Overlaps between candidate genes for local adaptation in indigenous African cattle as discussed in Ayalew et al. (15) and candidate genes under selection identified in this study. Table S6. Overlaps between candidate genes for local adaptation in indigenous African cattle as discussed in Kambal et al. (55) and candidate genes under selection identified in this study.

Additional file 2: Table S1. Breed-wise selection signatures. Position (chromosome, start, stop), maximum z-score, length and genes for selection signatures identified with iHS approach. Selection signatures were defined as peaks of the iHS statistic (z-scores) for each breed with at least one variant that exceeded the minimum top 0.01% z-score.

Additional file 3: Table S2. STRING-enriched terms of selection signatures. For each breed, terms (and their categories) which were significantly enriched by genes located in selection signatures are reported.

Additional file 4: Table S3. Regions of breed-wise selection signatures and their intersections between breeds. Intersections between selection signatures for all breeds were identified using the option ‘multiinter’ in bedtools and intersections within 50kb of each other were grouped into regions. ID, position (chromosome, start, stop), overlap information (number of selection signatures in region, number of breed overlaps, overlapping breeds), length and genes for identified regions are reported.

Additional file 5: Figure S3. Scatterplots for *F*_ST_ and absolute *cis*-eQTL effect size with regression line and Spearman correlation (“R”) are presented for each tissue.

Additional file 6: Table S4. Tissue-wise Wilcoxon test for distribution differences of the absolute *cis*-eQTL slopes between adaptive and non-adaptive variants. Variants belonging to the top 1% of global *F*_ST_ were considered as adaptive. Tissue, group (adaptive vs. non adaptive), variants per group, median absolute *cis*-eQTL, p-value and significance of the comparison are reported. For each tissue, the variant in the top 1% *F*_ST_ with highest *cis*-eQTL slope is also reported (with variant location, eGene, allele information, *F*_ST_ and eQTL slope).

Additional file 7: Table S5. Overlaps between selection signatures and QTL. Selection signatures were mapped to the Cattle QTL data base. Trait, position (chromosome, start, stop), QTL name, QTL ID, trait association and trait group for QTL that were located in selection signatures are reported for each breed.

Additional file 8: Figure S4. Number of QTL located within selection signatures for QTL trait groups.

## Data Availability

Table 1 contains the European Nucleotide Archive project accession codes of samples used in this study.
