# Supplementary material for: Insights into trait-association of selection signatures and adaptive eQTL in indigenous African cattle
Source: BMC Genomics. 2024 Oct 19;25:981. doi: 10.1186/s12864-024-10852-8 (PMC11490109; doi:10.1186/s12864-024-10852-8)
Supplement: Supplementary file 1 — Additional file 1: Figure S1. Principal component analysis of African cattle breeds. Eigenvectors for the first two principal components are plotted and the variances explained by the principal components are given in parentheses, with samples coloured by breed (abbreviations: ANK, Ankole; BAO, Baoule; BOR, Boran; DJA, Djakkore; GOBZ, Gobra; KEN, Kenana; NDA, N’dama; OGA, Ogaden). Figure S2. Admixture analysis of African cattle breeds. Proportion of genetic admixture for K =1, … ,5 of 65 African cattle samples. Breed abbreviations: ANK, Ankole; BAO, Baoule; BOR, Boran; DJA, Djakkore; GOBZ, Gobra; KEN, Kenana; NDA, N’dama; OGA, Ogaden. Table S5. Overlaps between candidate genes for local adaptation in indigenous African cattle as discussed in Ayalew et al. (15) and candidate genes under selection identified in this study. Table S6. Overlaps between candidate genes for local adaptation in indigenous African cattle as discussed in Kambal et al. (55) and candidate genes under selection identified in this study. [file 12864_2024_10852_MOESM1_ESM.docx]

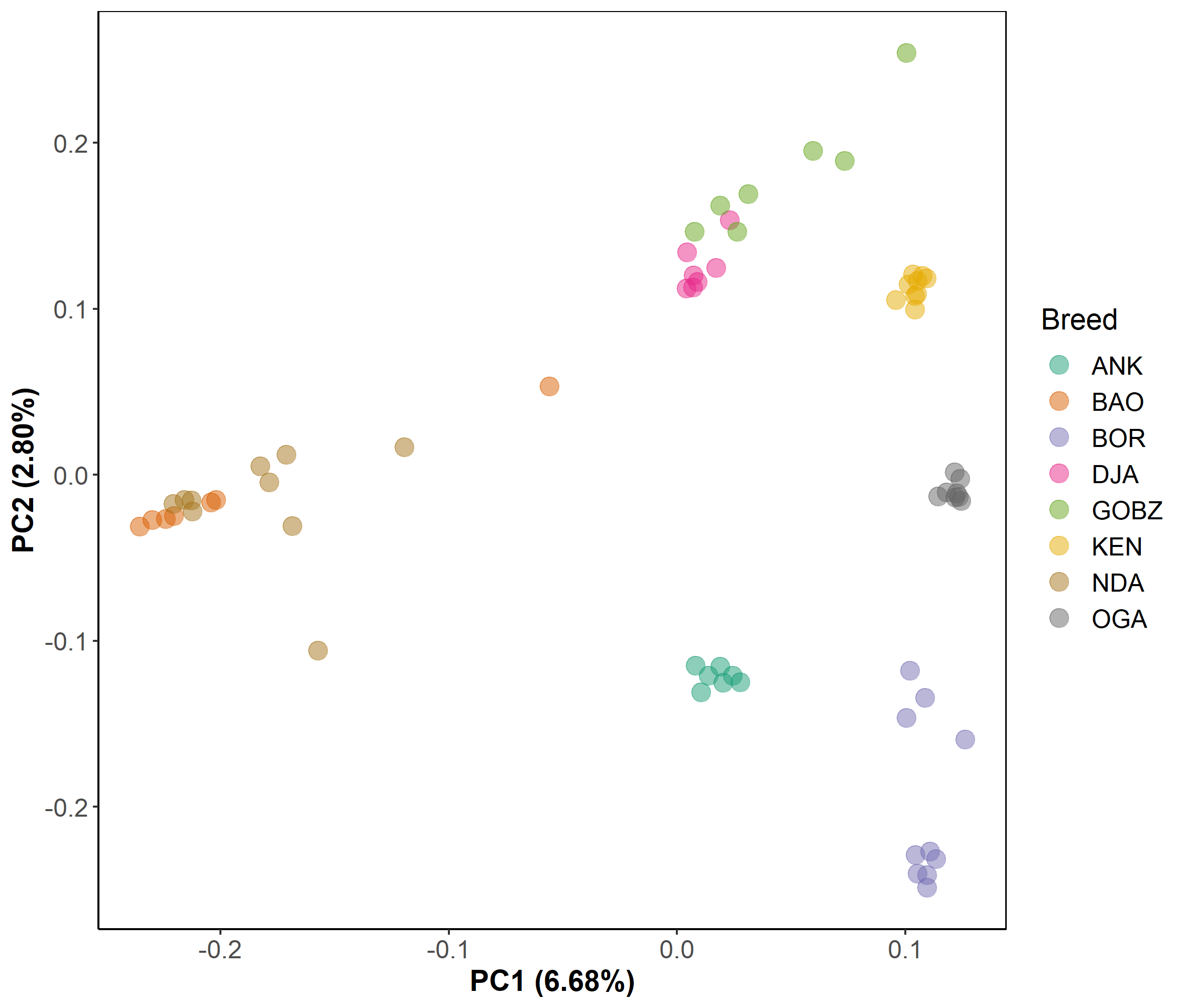


**Figure S1. Principal component analysis of African cattle breeds.** Eigenvectors for the first two principal components are plotted and the variances explained by the principal components are given in parentheses, with samples coloured by breed (abbreviations: ANK, Ankole; BAO, Baoule; BOR, Boran; DJA, Djakkore; GOBZ, Gobra; KEN, Kenana; NDA, N’dama; OGA, Ogaden).





**Figure S2. Admixture analysis of African cattle breeds.** Proportion of genetic admixture for K =1, … ,5 of 65 African cattle samples. Breed abbreviations: ANK, Ankole; BAO, Baoule; BOR, Boran; DJA, Djakkore; GOBZ, Gobra; KEN, Kenana; NDA, N’dama; OGA, Ogaden.

**Table S5. Overlaps between candidate genes for local adaptation in indigenous African cattle as discussed in Ayalew et al. (12) and candidate genes under selection identified in this study.**

| Gene | Data | Statistical method | Association | Breed^1^ | Reference | Breed^2^ |
| --- | --- | --- | --- | --- | --- | --- |
| *CSN3* | WGS | FST, XP-CLR, Pi(π) | Milk production | Kenana | (Asadollahpour Nanaei et al. 2020) | Kenana |
| *GPX5* | WGS | iHS, Rsb | Reproduction and fertility traits | Muturu | (Tijjani et al. 2019) | Djakkore |
| *PLA2G2A* | WGS | XP-CLR, XP-EHH | Intramuscular fat, involved in adipose metabolism and adipogenesis | Ankole | (Taye, Kim, et al. 2017; Taye, Lee, Caetano-Anolles, et al. 2017; Taye, Lee, Jeon, et al. 2017) | Gobra |
| *PPP1R14C* | Bovine HD array | CLR, iHS, Rsb | Trypanotolerance | Sheko | (Mekonnen et al. 2019) | Djakkore |
| *TRIM21* | Bovine SNP50 array | F_ST_ | Trypanotolerance | N’Dama, Baoulé, Somba, Nadoba, Lagune, Borgou | (Gautier et al. 2009) | Ankole |
| *WC1* | Bovine SNP50 array | F_ST_ | Spermatogenesis, ovulation rate, oestrus processes, testis and prostaglandin development | Afrikaner, Nguni, Drakensberger, Bonsmara | (Makina et al. 2015) | Gobra, Djakkore, N'Dama, Ogaden |

^1^ analysed breeds in cited study

^2^ analysed breeds in our study

**Table S6. Overlaps between candidate genes for local adaptation in indigenous African cattle as discussed in Kambal et al. (53) and candidate genes under selection identified in this study.**

| Gene | Data | Statistical method | Description | Reference | Breed^2^ |
| --- | --- | --- | --- | --- | --- |
| *GZMM* | Bovine SNP50 array | iHS, XP-EHH, Rsb | Major candidate gene | (Ben-Jemaa et al. 2020) | Ogaden |
| *POLR3B* | Bovine SNP50 array | iHS, Rsb, CLR | Resistance to vector borne diseases | (Ben-Jemaa et al. 2020; Kim et al. 2020; Mauki et al. 2022; Mekonnen et al. 2019; Taye et al. 2018) | Kenana |
| *PPP1R14C* | Bovine SNP50 array | iHS, Rsb, CLR | Resistance to vector borne diseases | (Ben-Jemaa et al. 2020; Kim et al. 2020; Mauki et al. 2022; Mekonnen et al. 2019; Taye et al. 2018) | Djakkore |
| *TRIM21* | Bovine SNP50 array | BF, F_ST_ | Major candidate gene | (Gautier et al. 2009) | Ankole |

^2^ analysed breeds in our study
