# Supplementary figures and images for: Insights into trait-association of selection signatures and adaptive eQTL in indigenous African cattle
Source: BMC Genomics. 2024 Oct 19;25:981. doi: 10.1186/s12864-024-10852-8 (PMC11490109; doi:10.1186/s12864-024-10852-8)

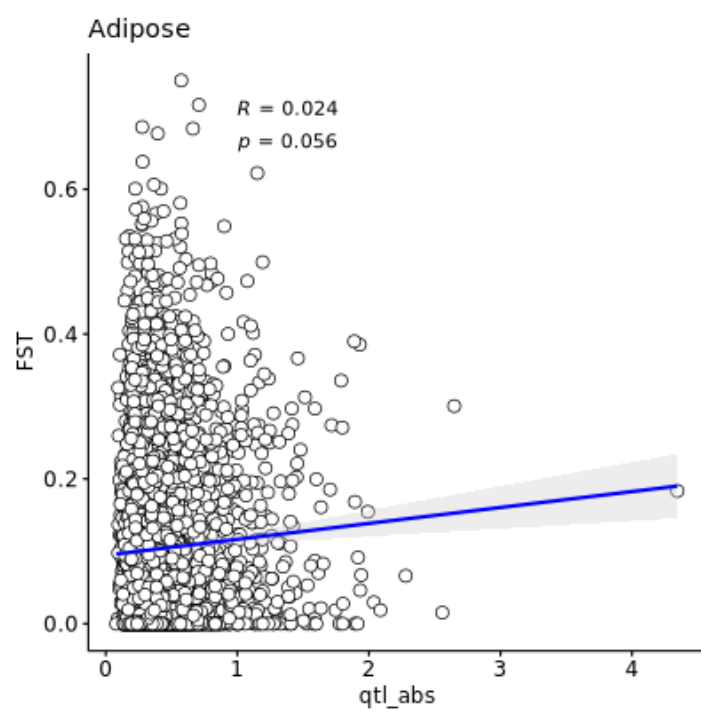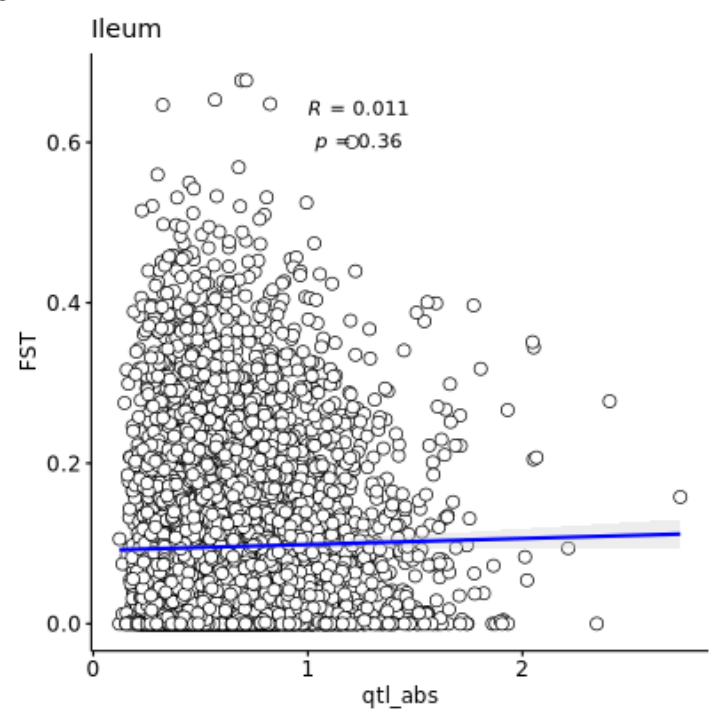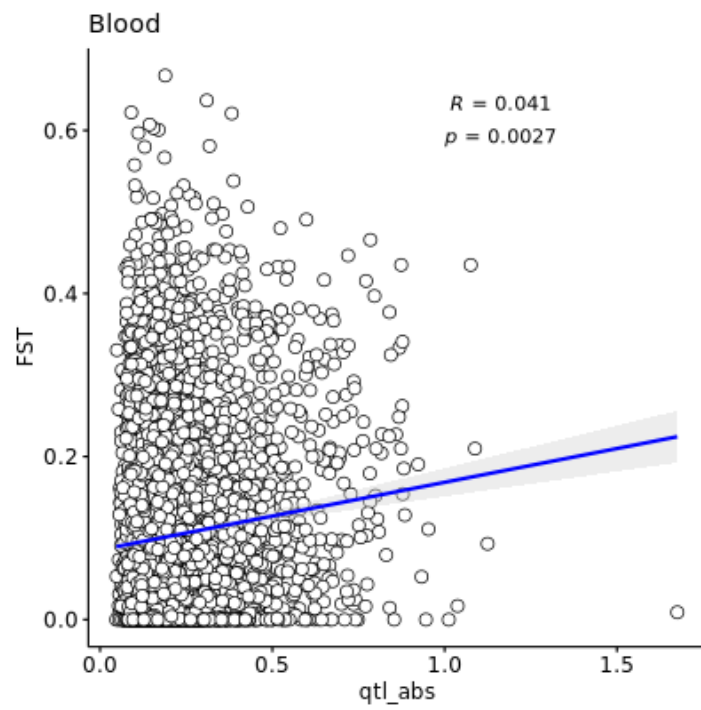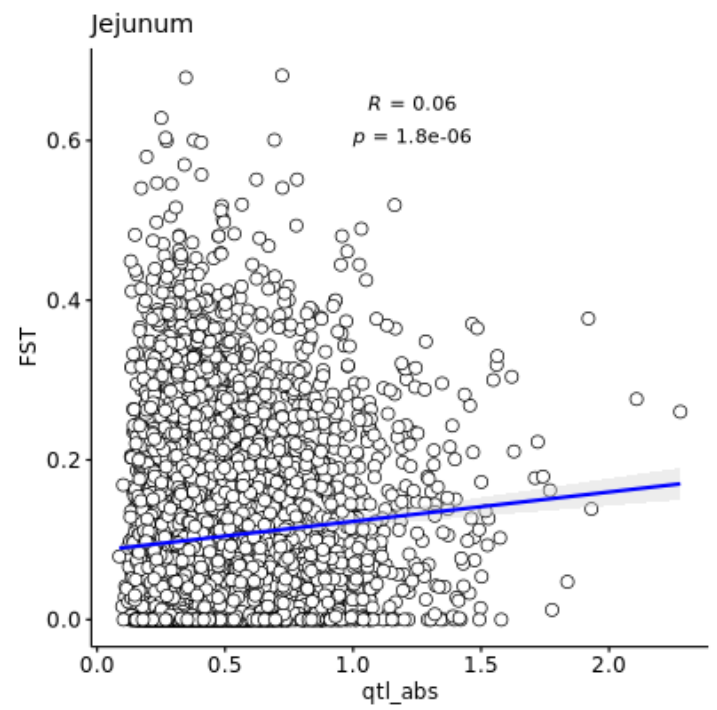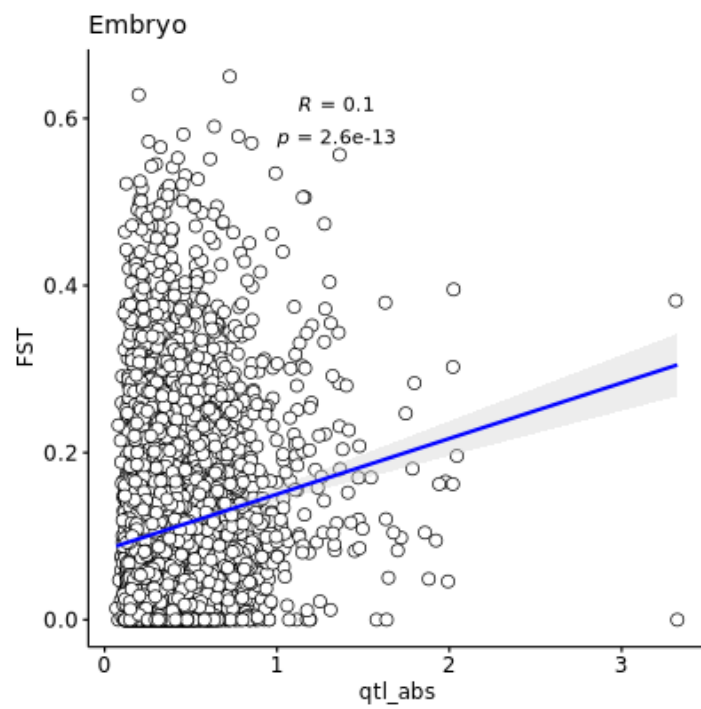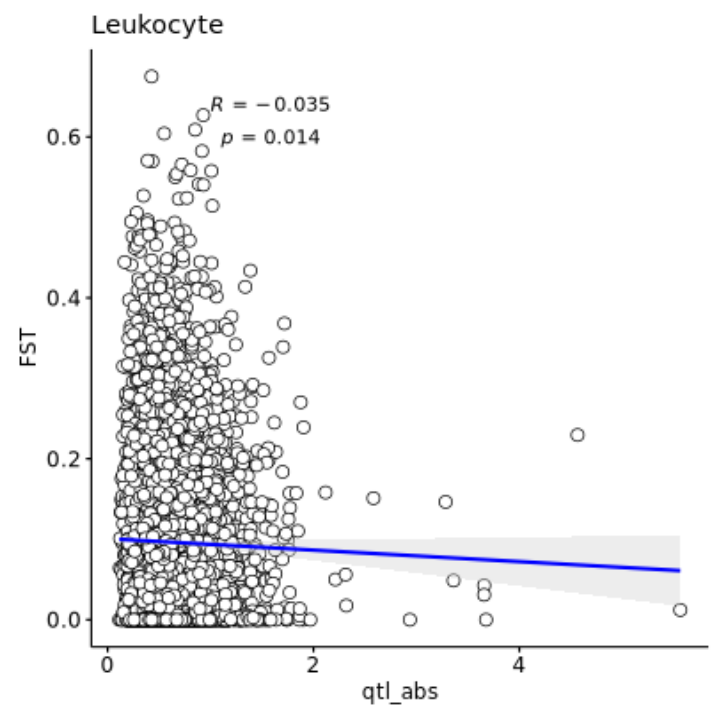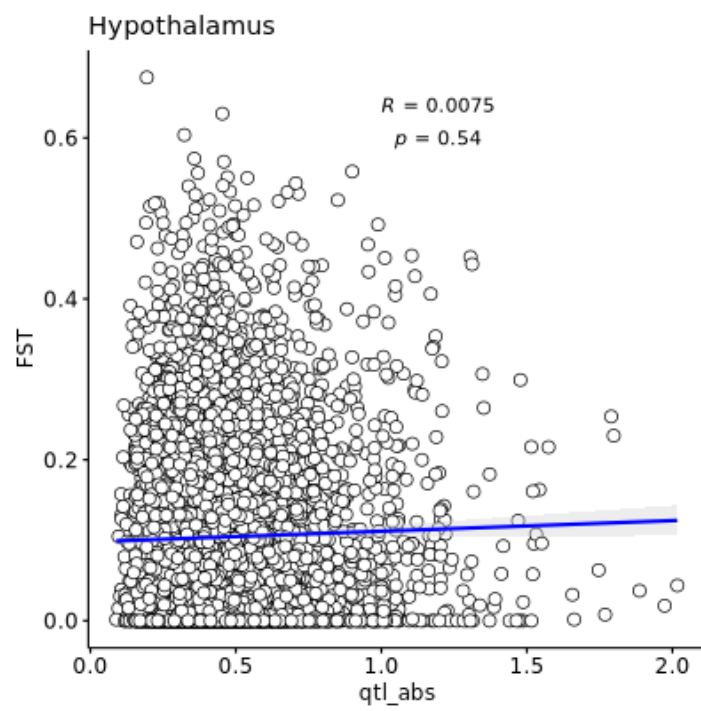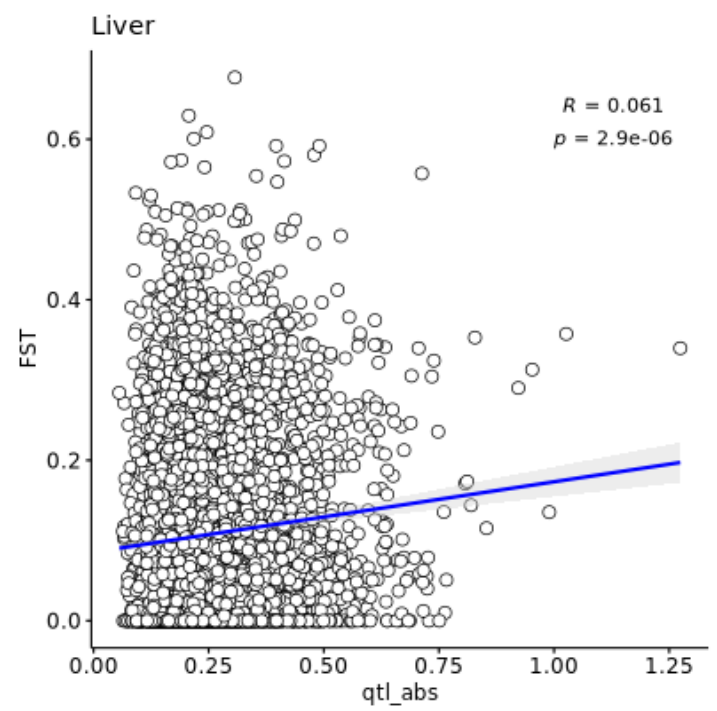

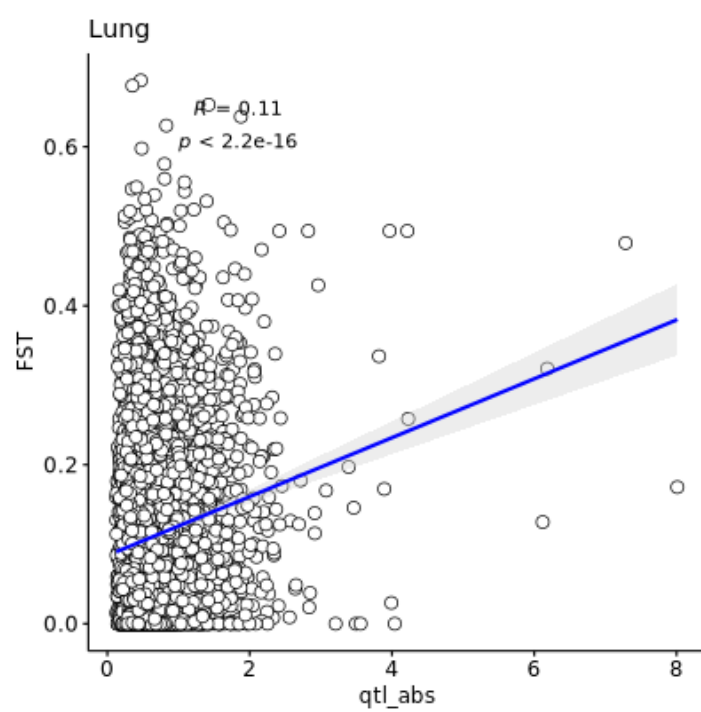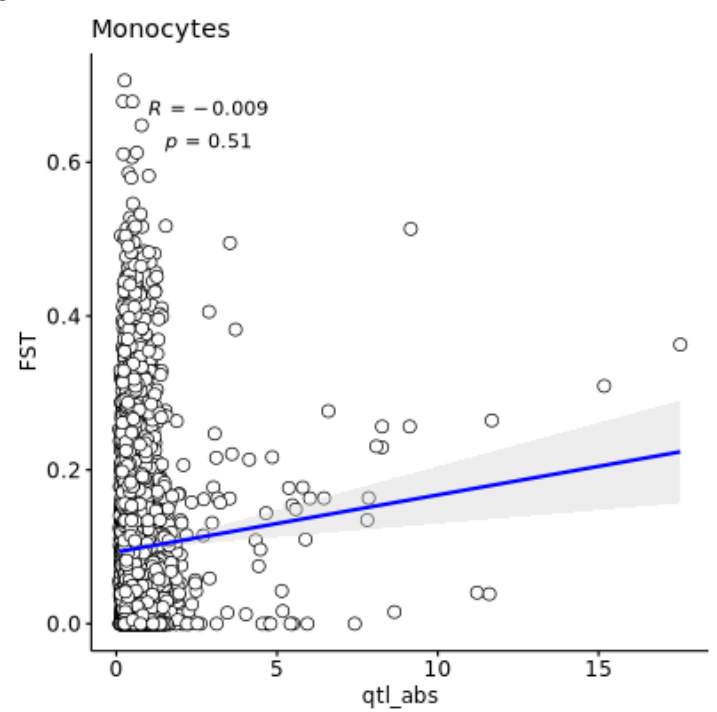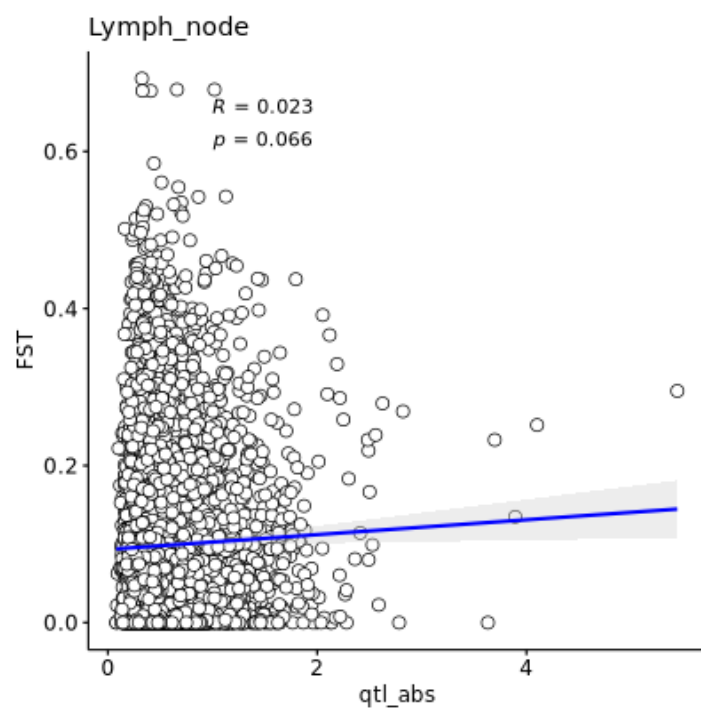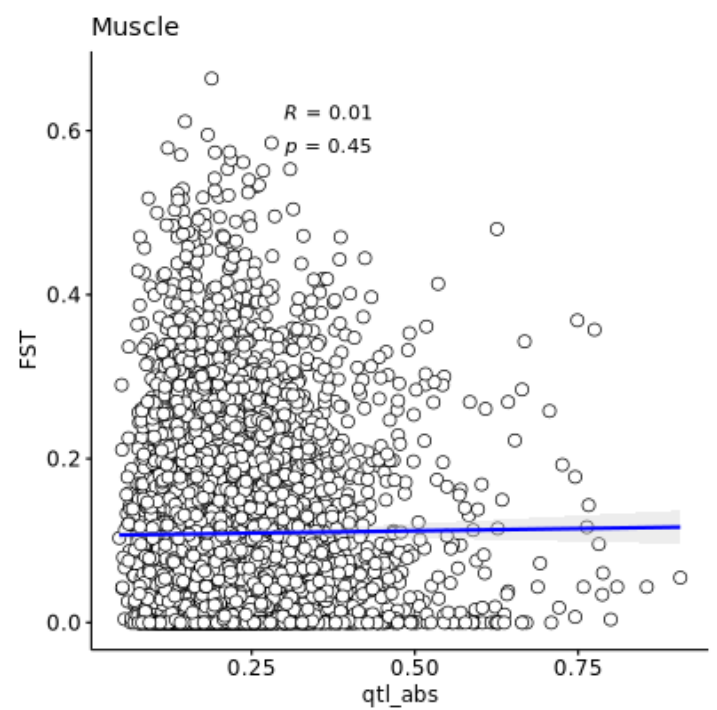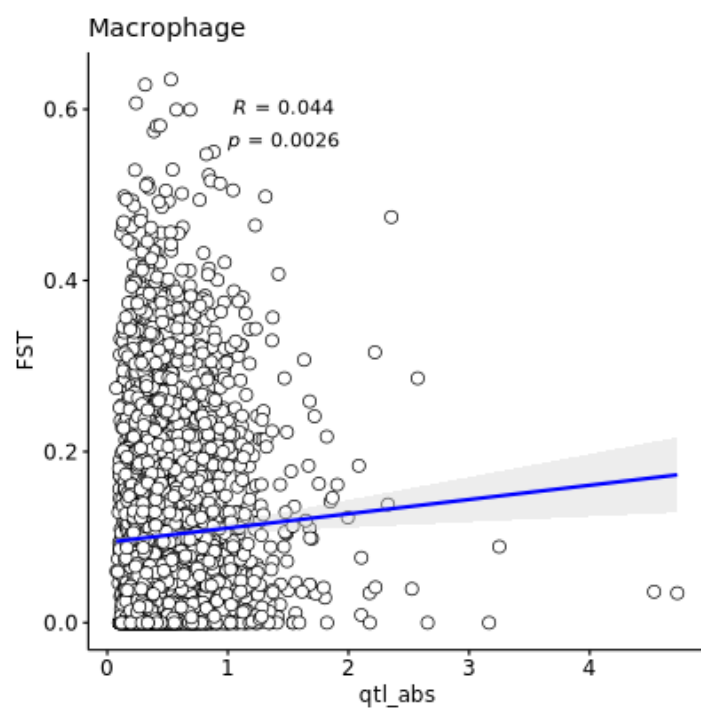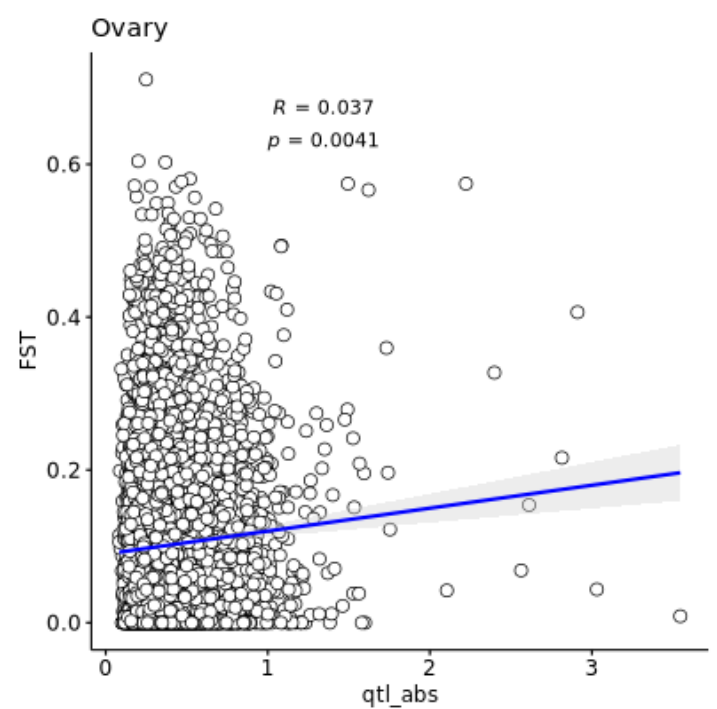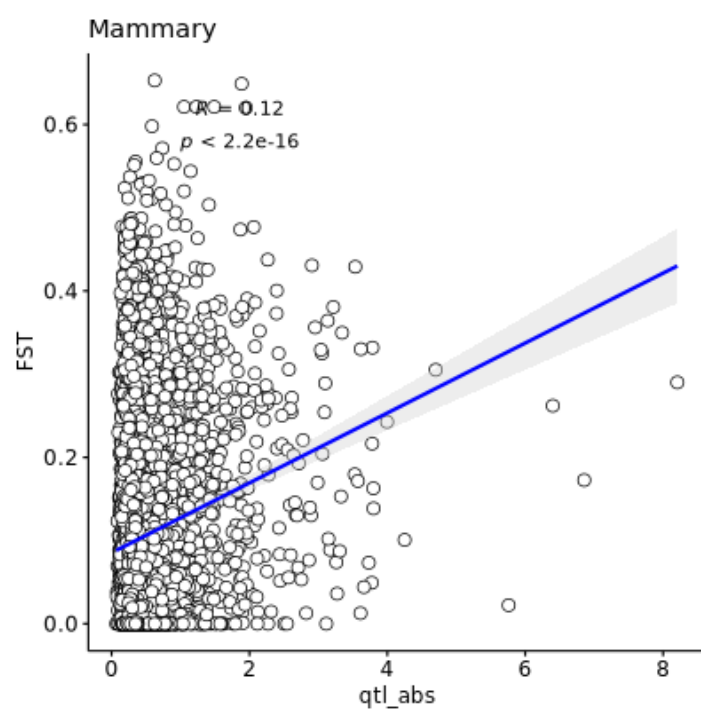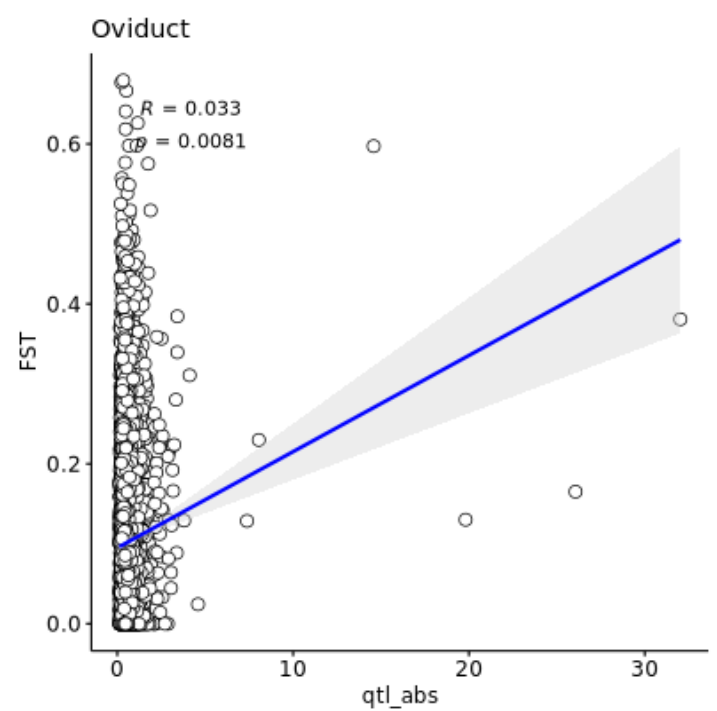

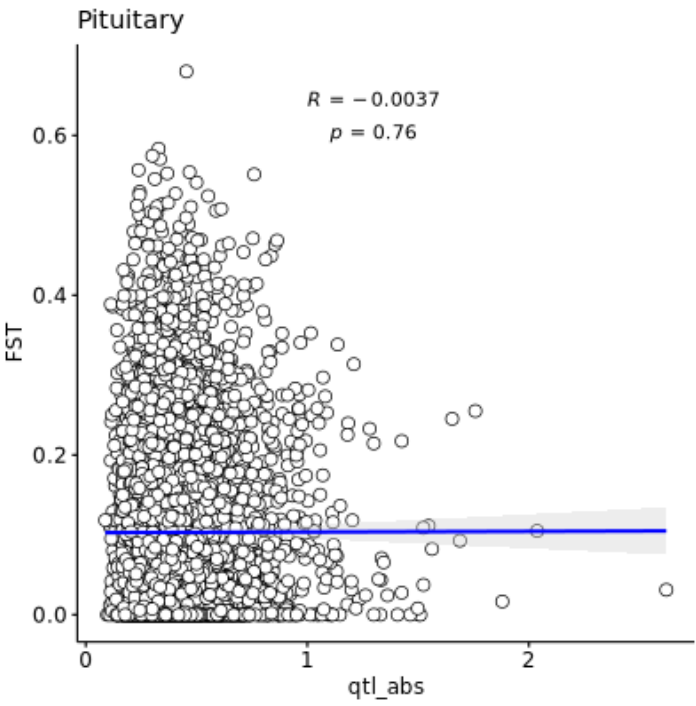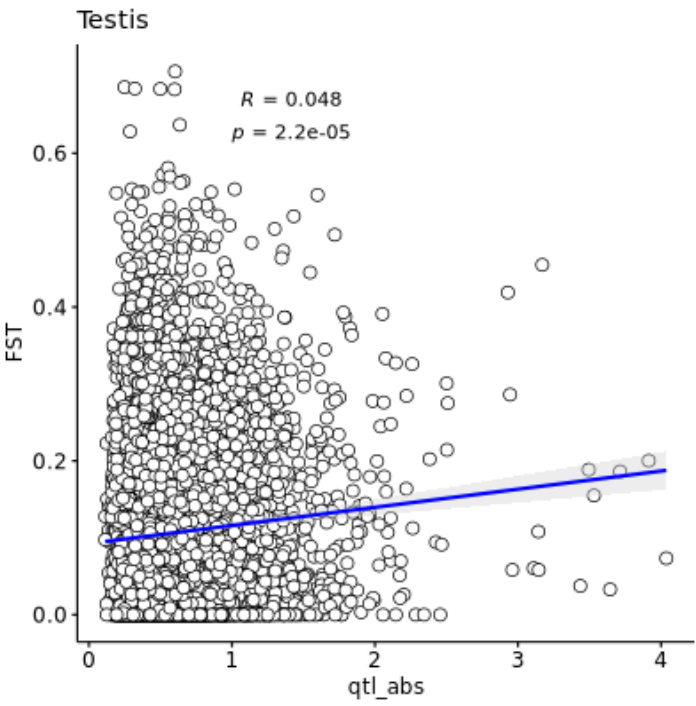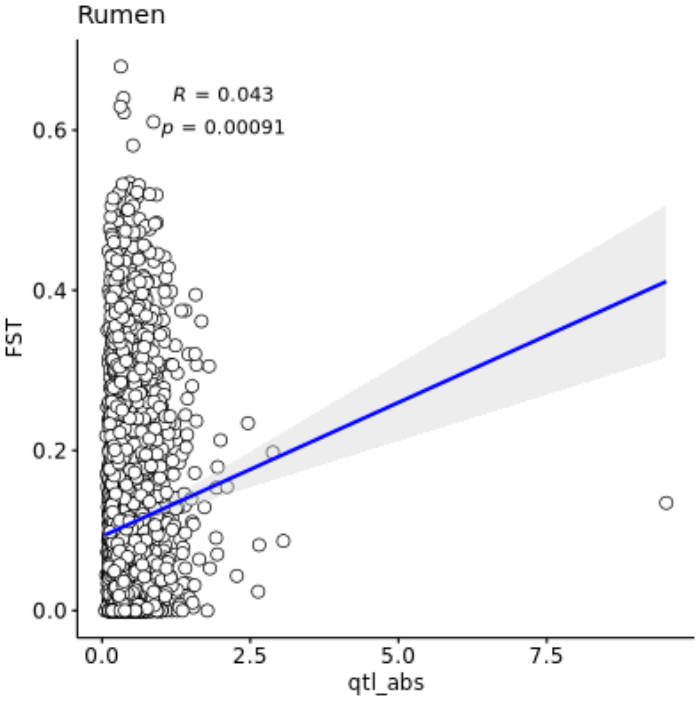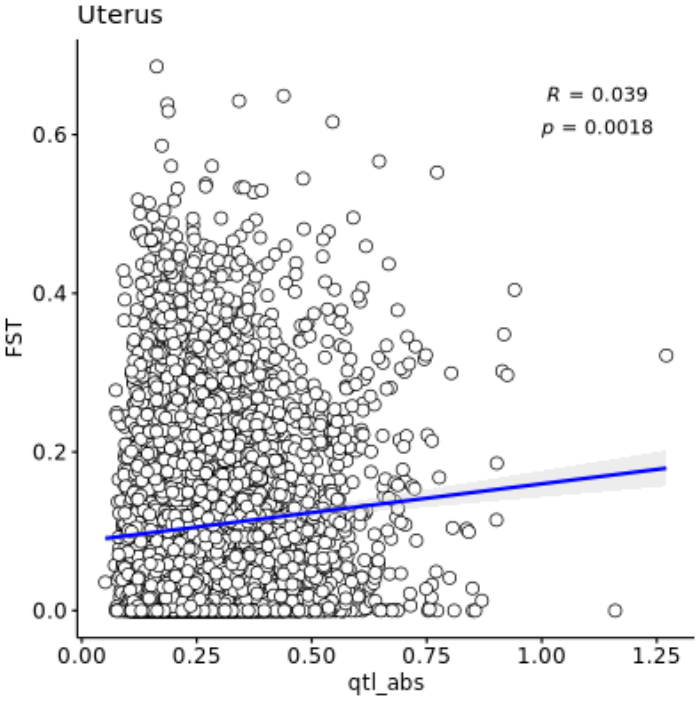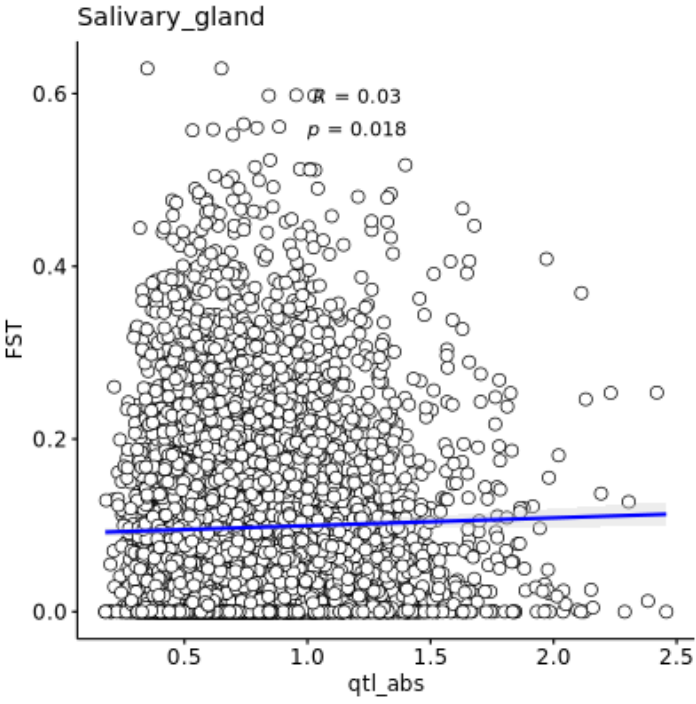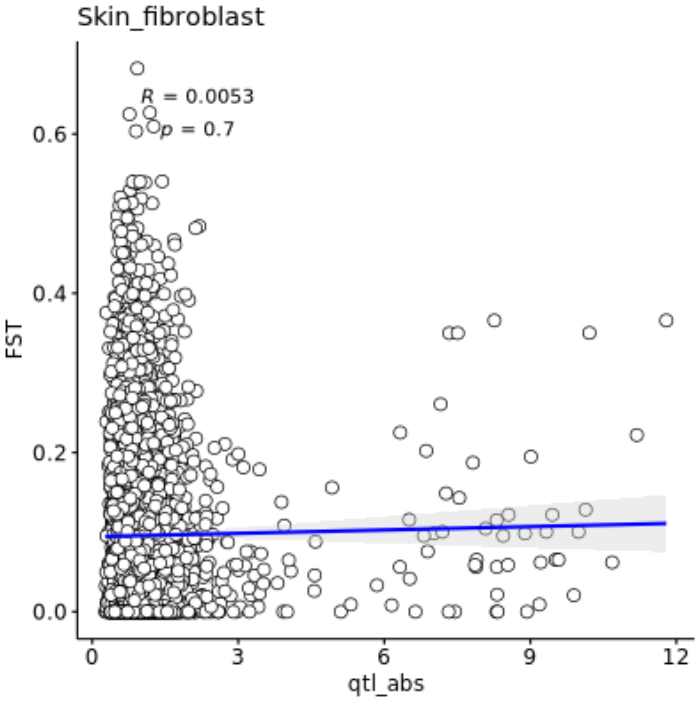

Supplement: Supplementary file 5 — Additional file 5: Figure S3. Scatterplots for FST and absolute cis-eQTL effect size with regression line and Spearman correlation (“R”) are presented for each tissue. [file 12864_2024_10852_MOESM5_ESM.pdf]

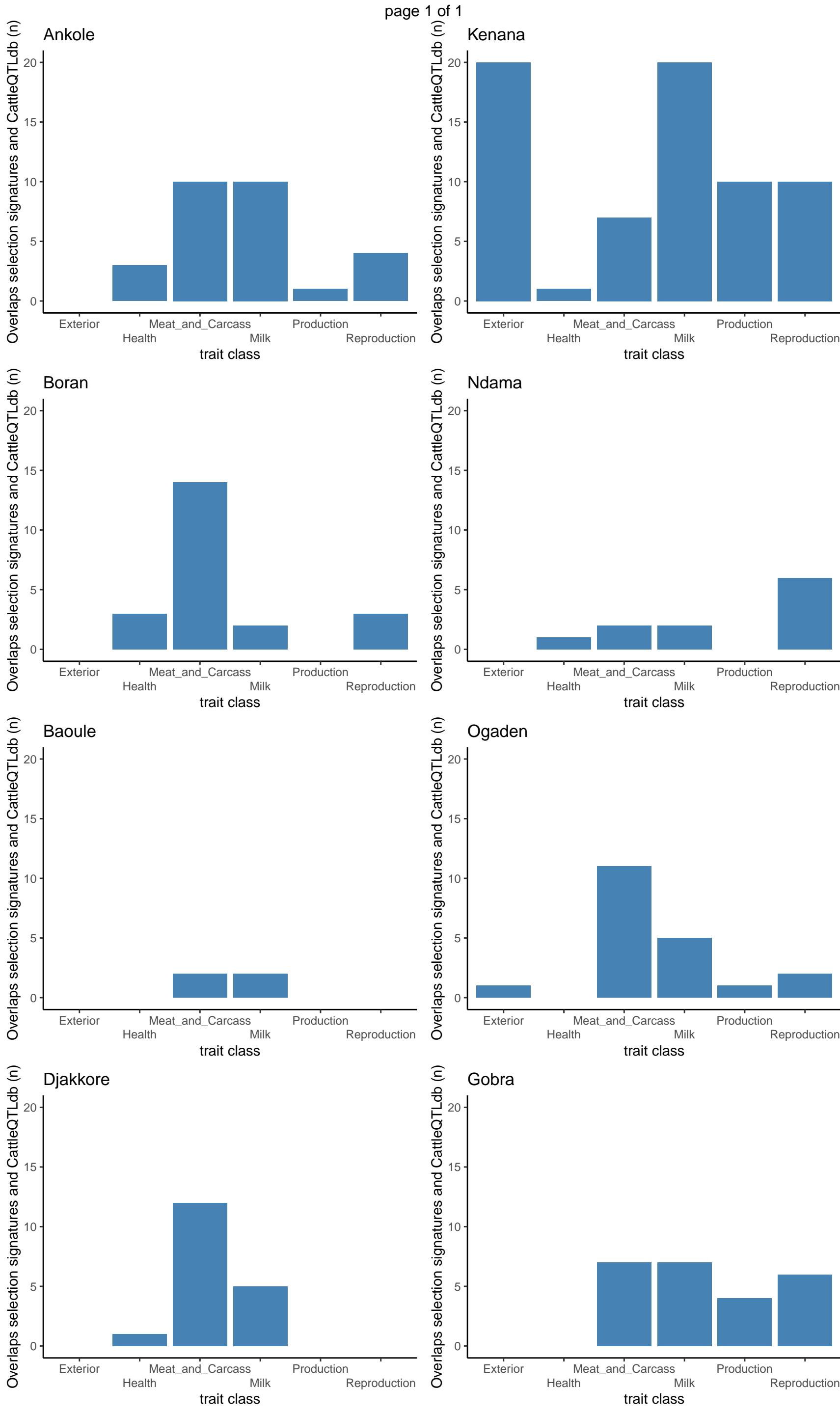

Supplement: Supplementary file 8 — Additional file 8: Figure S4. Number of QTL located within selection signatures for QTL trait groups. [file 12864_2024_10852_MOESM8_ESM.pdf]
